# Proton Irradiation on Halide Perovskites: Numerical Calculations

**DOI:** 10.3390/nano14010001

**Published:** 2023-12-19

**Authors:** Alexandra V. Rasmetyeva, Stepan S. Zyryanov, Ivan E. Novoselov, Andrey I. Kukharenko, Efrem V. Makarov, Seif O. Cholakh, Ernst Z. Kurmaev, Ivan S. Zhidkov

**Affiliations:** 1 Institute of Physics and Technology, Ural Federal University, Mira 19 Street, 620002 Yekaterinburg, Russia; 2 M.N. Mikheev Institute of Metal Physics of Ural Branch of Russian Academy of Sciences, S. Kovalevskoi 18 Street, 620108 Yekaterinburg, Russia; 3 Institute of Electrophysics of Ural Branch of Russian Academy of Sciences, Amundsena 106 Street, 620110 Yekaterinburg, Russia

**Keywords:** hybrid perovskites, proton irradiation, stability, SRIM, SCAPS

## Abstract

The results of numerical SRIM and SCAPS calculations for the ionization, displacement and heating of hybrid perovskites under the influence of protons (E = 0.15, 3.0 and 18 MeV) are presented and show that the lowest transfer energy is demonstrated by the MAPbI_3_, FAPbBr_3_ and FAPbI_3_ compounds, which represent the greatest potential for use as solar cells in space devices. On the other hand, it is found that perovskite compositions containing FA and Cs and with mixed cations are the most stable from the point of view of the formation of vacancies and phonons and are also promising as radiation-resistant materials with respect to powerful proton fluxes. Taking into account the lateral distribution of proton tracks showed that, at an energy level of several MeV, the release of their energy can be considered uniform over the depth and area of the entire solar cell, suggesting that the simple protection by plastic films from the low-energy protons is sufficient.

## 1. Introduction

Recently, there has been increasing interest in the use of halide perovskites for space photovoltaic technology for several reasons. First of all, it was found that irradiation with electrons and protons in the MeV mode significantly deteriorates the properties of inorganic electronic devices based on Si, InGaP, GaAs or Ge [1], and the search for radiation-resistant solar cells has become an urgent task for space applications. The choice of perovskite solar cells was supported by the fact that the first experiments already showed that the radiation resistance of MAPbI_3_ exceeds the radiation resistance of c-Si by almost three orders of magnitude [2]. An important argument in favor of perovskite solar cells was that space photovoltaic devices requiring the radiation and temperature stability remove such strong restrictions as a long (25 years) operation, humidity and oxygen atmosphere, which represent a bottleneck for operation in terrestrial conditions [3]. Another reason favoring space applications of perovskite elements is their high efficiency (PCE = 26.1% [4]) and impressive radiation stability achieved in the last few years [5,6,7]. Unlike the most terrestrial photovoltaic applications, where the W/USD ratio is considered the most important factor; for space solar modules, the W/kg ratio is the key parameter that sets perovskite solar cells apart from their III-V solar cell counterparts. It was recently demonstrated that, after 10 months on the International Space Station, perovskite solar panels showed good stability in regard to their electrophysical properties [8]. However, space tests do not allow us to assess the nature of the changes taking place and, therefore, to develop ways to solve emerging instabilities. According to [6], the thin perovskite films can withstand irradiation fluences exceeding 10^15^ p/cm^2^ which is much higher than that found for the conventional III–V space solar cells without encapsulation, as these require an additional cover glass [9]. The experimental testing of solar cells for space applications in terrestrial conditions is quite a difficult task for the following reasons. As a rule, it is limited to only a few discrete energies of particles (electrons/protons), whereas cosmic radiation covers a wide range of energies and fluences. In addition, the accelerated degradation tests require the high particle fluxes necessary for modeling the general radiation doses during the long-term space flights. This may cause thermal and other changes in test samples which would not occur in the real space conditions. And, finally, the radiation tests are not widely available, and the testing process itself is slow, expensive, limits options sampling and does not allow each sample to be characterized for a full range of particle types and energies. In this regard, the development of numerical modeling, which, in combination with limited experimental tests, can give the optimal results, is of great importance.

An attempt was made to determine which proton exposure regimes are most appropriate for assessing the radiation resistance of halide perovskites. It has been shown that protons with energies of 0.5–0.15 MeV have the greatest negative effects on perovskites [10]. However, under the conditions of terrestrial tests, the most easily obtained energy range for protons still remains 3–68 MeV [3] because the dense beams of protons with such energies are widely used in practice with accelerator technology. On the other hand, protons of 0.5–0.15 MeV under terrestrial conditions can be obtained primarily as a result of decay reactions. However, in this case, we get significantly lower flows, meaning that more exposure time will be required to study the stability of perovskite solar cells (PSCs) in practice. In addition, the uniform distribution of all protons over the entire area of the solar cell is usually not taken into account during the possible irradiation of the PSC at the accelerator. In this work, using the SRIM method [11], we not only assessed the effect of protons of different energy ranges on the APbX3 perovskite but also analyzed the radiation resistance of a wide range of compositions with changes in both A (MA–CH_3_NH_3_, FA–CH_2_(NH_2_)_2_ and Cs) and X component (I and Br). We pay special attention not only to the distribution of displacements and ionization but also to the possible formation of phonons (heating) of PSCs during irradiation. In addition, using the SRIM simulation, we also evaluated the defect formation in the PSC on its I–V characteristics and external quantum efficiency (EQE).

## 2. Theory and Calculation Details

The heavy charged particles interact mainly with electrons of atomic shells, causing the ionization of atoms. Passing through matter, a charged particle makes tens of thousands of collisions, gradually losing energy. The stopping power of a substance can be characterized by the value of the specific energy losses, dE/dx, where dE is the energy lost by a particle in a layer of substance of thickness, dx. If the energy of a charged particle is lost to ionize the matrix, then we speak of specific ionization losses. The specific energy losses increase with the decreasing particle energy and especially sharply before stopping in matter (Bragg peak).

Let us consider a heavy nonrelativistic charged particle with charge (ze) and speed (v), flying along the x-axis at a distance (b) from the electron (Supplementary Figure S1).

The maximum force of interaction at the moment of the closest approach of particles is equal to F = ze^2^⁄b^2^. If the interaction time is taken to be approximately equal to Δt ≈ 2 b/v, then the momentum transferred to the electron is Δp ≈ FΔt = 2 ze^2^/bv, and the energy transferred to it is ΔE ≈ (Δp)^2^/2 m_e_ = 2 z^2^ e^4^/m_e_v^2^b^2^ (m_e_, the electron mass). A volume element, dV, contains the number of electrons, dN = n_e_dV (n_e_, the electron density). Since dV = 2πb∙db∙dx, the total energy, dE, transferred to these electrons by the particle is given by the following expression:

dE = ∆E × dN = 4π∙(n_e_ × z^2^ × e^4^)/(m_e_ × v^2^) × (db/b) × dx. (1)

Integrating over b from b_min_ to b_max_, we obtain the value of specific ionization losses:

dE/dx =∙(n_e_ × z^2^ × e^4^)/(m_e_ × v^2^) × ln(b_max_/b_min_). (2)

At b_max_→∞ and b_min_→0, the integral diverges. In reality, the value of b_max_ is limited by the fact that, with a large b and small E, the atomic electron can no longer be considered free, since the interaction energy will be comparable to the ionization potential, and with a sufficiently large b, the transferred energy may be insufficient to excite the atom. The restriction on the lower limit of integration, b_min_, is due to the fact that the maximum energy can be transferred to an electron by a heavy nonrelativistic charged particle, ΔE_max_ = m_e_∙v^2^.

The specific ionization energy losses for heavy charged particles at energies of E ≪ (Mc)^2^/m_e_ (E and M, kinetic energy and mass of the particle) are calculated as follows:

(dE/dx)_ioniz_ = −4π × (z^2^/β^2^) × n_e_ × r_0_^2^ × m_e_ × c^2^ × [ln(2m_e_ × c^2^ × β^2^)/Ī) − ln(1 − β^2^) − β^2^], (3)

where m_e_ is the electron mass; c is the speed of light; β = v/c; v is the particle speed; z is the particle charge in units of positron charge; n_e_ is the electron density of the substance; Ī is the average ionization potential of the atoms of the substance of the medium through which the particle passes, Ī = 13.5∙Z, where Z is the charge of the nuclei of the medium in units of positron charge; and r_0_ = e^2^/(m_e_∙c^2^) = 2.818 × 10^−13^ cm is the classical electron radius.

Considering that the electron density of a substance is n_e_ = Zn, where n_e_ is the density of nuclei of the substance, and Z is the charge of nuclei in units of positron charge, we can express ne in terms of the parameters of the medium: n_e_ = Zn = Z × (ρN_A_)/A (N_A_ is Avogadro’s number, A is the mass number of nuclei of the medium, and ρ is the density of the medium in g/cm^3^). Then, the formula for the specific ionization losses of heavy particles is transformed into a form more convenient for calculations [12]:

−dE/dx = 3.1 × 10^5^ × (Z × z^2^ × ρ)/(A × β^2^) × (11.2 + ln(β^2^/Z(1 − β^2^)) − β^2^). (4)

These calculations and the resulting formulas for electrons are also valid for our case, that is, for protons.

The various computer simulation programs are used to calculate the range of ions in solids. The most popular is the SRIM software package 2013, which, along with the ability to calculate ranges, allows one to obtain other important information, such as the distribution of vacancies in the target, recoil atoms, etc. [13,14,15]. SRIM is a program for the transport of ions in non-crystalline matter, using the Monte Carlo method, without taking into account inelastic nuclear reactions. The program simulates all processes of interaction between an incident particle and target atoms. It is possible to set the kinetic energy of ions in a wide range up to 2 GeV, simulate complex target substances consisting of several layers (up to 8), etc. The program provides a high-accuracy modeling of ionization energy losses of the particles in matter, the energy spectra of recoil atoms, three-dimensional trajectories of particles considering multiple collisions with atoms and the processes of implantation of ionic impurities into matter, and it has a number of other options. TRIM (Transport of Ions in Matter, part of the SRIM package) is a program with a wide range of functions that is capable of calculating both the final three-dimensional distribution of ions and all kinetic phenomena associated with the ion energy loss: target damage, sputtering, ionization and phonon generation in complex multicomponent targets.

**Figure 1:**
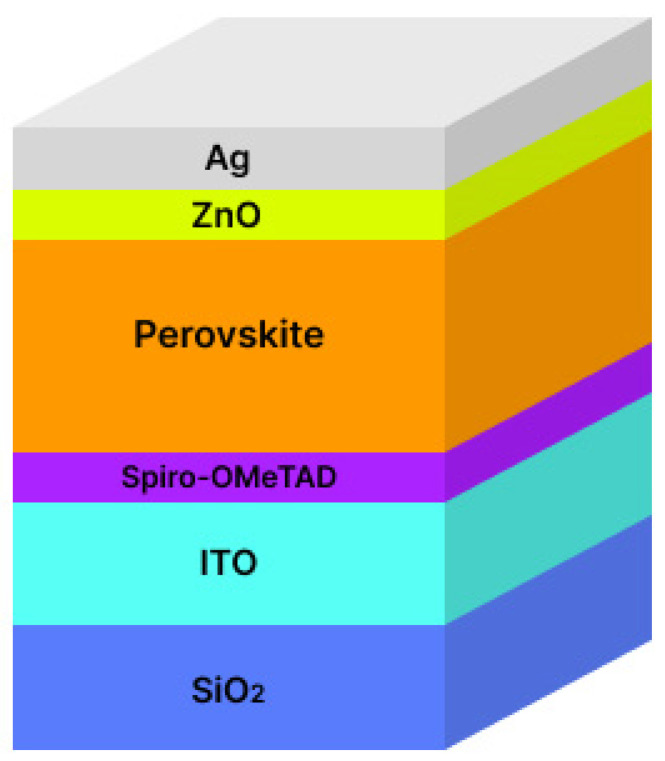
The structure of solar cells used for calculations by SRIM.

The effect of protons on a solar cell of the following architecture was simulated: Ag (100 nm)/ZnO (80 nm)/perovskite (500 nm)/Spiro-OMeTAD (80 nm)/ITO (200 nm)/SiO_2_ (500 μm). The structure used for calculations is presented in Figure 1. The inputs of proton beams with energies of 150 keV, 3 MeV and 18 MeV were considered from the side of the opaque electrode. A simulation was carried out for the influence of 10,000 protons flying into one infinitesimal point, which can be considered very large fluences, since typical fluxes in orbit are of the order of 10^12^–10^14^ p^+^/cm^2^.

SCAPS 3.3.10 (a Solar Cell Capacitance Simulator) is a one-dimensional solar cell simulation program developed at the Department of Electronics and Information Systems (ELIS) of the University of Gent, Belgium [16]. It allows us to evaluate the parameters of a solar cell depending on both the composition and structure of the layers (material, presence of defects, types of defects, etc.) and external conditions (illumination, temperature, etc.). The following conditions were used for our calculations: spectrum AM, 1.5 G; 1 sun; transmission, 100%; and spectrum cutoff—no. Solar cells’ architectures were the same as for SRIM calculations. We varied the density of defects in solar cell materials to simulate the effects of protons. Five different defect densities were used: original, 10 times original, 100 times, 1000 times and 10,000 times. Find the detailed parameters of materials for modeling in Supplementary Tables S1 and S2, taken from References [17,18,19,20,21,22,23].

## 3. Results and Discussions

### 3.1. SRIM Calculations

Figure 2, Figure 3, Figure 4 and Figure 5 and Supplementary Figures S2–S13 present the calculation data for a perovskite layer in the SRIM 2013 package. The most important conclusion that arises from the initial analysis of all figures is the noticeably lower energy costs of protons when colliding with perovskite atoms, both for the formation of vacancies/displacements and for the ionization and heating (formation of phonons). This applies both to comparisons between all layers of a solar cell and when comparing fast protons with low energy ones.

If we consider the perovskite layer separately from the rest of the cell, it is noticeable that protons interact most effectively with the halide (iodine or bromine) atoms. This manifests itself both in the transfer of energy to halide atoms and, as an example, in the formation of iodine vacancies. This fact is one of the most important from the point of view of the stability PSC in space, since iodine vacancies are the seeds for the further thermal and photochemical degradation of perovskite [24,25]. Thus, the passivation of halide vacancies is extremely important for the stable operation of a perovskite solar cell in space. It should be considered that the methods existing today are usually associated with doping by organic molecules along the grain boundaries of the perovskite film [26,27]. The vacancies formed as a result of the proton beam will be uniformly distributed throughout the depth and lateral area of the entire perovskite film, which follows from the distribution of vacancies and proton tracks in the sample (Figure 4 and Figure 6). Thus, for use in the space, it is necessary to find other ways to passivate the halide vacancies.

On the other hand, if we consider the entire solar cell, it is noteworthy that the primary displaced atoms spend several orders of magnitude more on ionization than the primary proton beam (Figure 3). Also, the primary displaced atoms make a significant contribution to heating due to the transfer of their energy to phonons (Figure 4). If we are talking about a perovskite layer, then everything here will again be determined primarily by the displaced halide ions. However, for the entire PSC as a whole, it should be noted that ITO absorbs several times more energy from the primary beam per unit length than the perovskite layer. The same applies to the back electrode (in our case, silver, but similar conclusions are valid for gold and copper). Thus, both electrodes will make a significant contribution to heating (Figure 5). At the same time, an important consequence of the significant absorption of proton beam energy by electrodes is the fact that they can also perform a protective function for the perovskite layer, while having a small thickness, and therefore enclosing the total mass of the solar cell, which is extremely important for space applications [28]. Thus, the rational engineering of solar cell architecture should be given great attention.

According to the latest literature data, it is recommended to use the protons with energies of 0.05–0.15 MeV for testing perovskite solar cells [7,10]. At the same time, although the transfer of energy to materials through all channels when irradiated with protons of 0.15 MeV is 5–10 times higher than with a proton energy of 3 MeV, the acquisition of significant fluences requires much more time. In addition, irradiation with protons of 0.15 MeV also leads to much more significant heating (Supplementary Figures S5, S9 and S13) than in the case of protons with an energy of 3 MeV, which can promote thermal degradation and will not allow the radiation effect of protons to be studied in a laboratory experiment. 

Considering that protons with an energy of 150 keV are easily shielded by increasing the thickness of the back electrode (to approximately 2 μm), we believe that it is advisable to conduct the laboratory experiments by using proton energies of 2–3 MeV. In addition, it is known that if we exclude from consideration the stage of final slowdown of particles in matter, the slowdown of both fast particles and the transfer of energy to electrons leads to the same results as the slowdown of β-particles or the scattering of γ-rays. The formation of electrons occurs with an energy significantly exceeding the energy chemical bonds in the lattice This circumstance makes it possible to study the processes of the transformation of matter under the influence of β- and γ-rays under accelerated simulation conditions, replacing irradiation with β- and γ-rays by bombarding samples with proton beams [29].

Among all perovskite compositions, the least energy transfer from protons to all channels (ionization, displacement and heating) is demonstrated by the MAPbI_3_, FAPbBr_3_ and FAPbI_3_ compounds (Supplementary Figures S2–S13). This is also supported by the results of gamma-ray and electron resistance studies, which show that the FAPbI_3_ has great potential for use in space [30].

As mentioned above, the most significant contribution to the formation of phonons is made by the displaced atoms, and not by primary ions (protons). Thus, the formation of vacancies can also indicate not only the formation of defects, which can act as charge traps and recombination centers, but also a greater contribution to heating. At the same time, the compositions containing Cs are the most stable from the point of view of the formation of vacancies and phonons. Thus, the compositions with partial mixing of FA and Cs in the A-cation site also seem promising from the point of view of radiation resistance with respect to powerful proton fluxes. 

To test this hypothesis, we carried out simulations for mixed A-cation perovskite Cs_0.12_FA_0.88_PbI_3_, which has previously shown good results for gamma-ray resistance. A comparison with MAPbI_3_ and FAPbI_3_ indeed indicates that, in the case of Cs_0.12_FA_0.88_PbI_3_, less energy is transferred by the displaced atom, and fewer vacancies are sampled (Supplementary Figures S14 and S15).

At the same time, all our conclusions do not take into account the lateral distribution of the incident beam. Figure 6 shows the lateral depth distributions of proton tracks. We also observe that the largest part of protons at any energy will give up a significant part of their energy in charge transport layers (CTLs) and electrodes. 

Let us consider in detail the influence of the proton energy on their trajectories in the PSC (Supplementary Figures S16–S18). It is obvious that, in comparison with fast protons, in the case of an energy of 0.15 MeV, a significant part of them will remain in the perovskite layer. However, even in this case, they will be most significantly slowed down in glass, which makes protection from such protons quite simple. Another interesting phenomenon is the partial backscattering of protons with energies of 0.15 MeV and 3 MeV at the interfaces between the layers. In this case, backscattered protons will lose a significant part of their energy near the interface. This fact explains the larger number of vacancies, ionized atoms and phonons at the far perovskite/CTL interface. Note that, for 18 MeV protons, this effect is practically absent, which means that the distribution of defects in the perovskite layer turns out to be more uniform.

On the other hand, the fast neutron beam (3 MeV and 18 MeV) is more uniform in the transverse view (right panels in Supplementary Figures S16–S18). Therefore, for protons with an energy of several MeV, the release of their energy can be considered more uniform over the depth and area of the entire solar cell. Based on these results and fairly simple protection from low-energy protons, such as transparent plastic films, we conclude that it is necessary to conduct ground-based tests with protons with energies of several MeV.

### 3.2. Calculation of Activation of Irradiated Materials (Inelastic Nuclear Reactions) and Specific Ionization Losses of Proton Energy in Perovskite Samples

Under the influence of protons, the following nuclear reactions occur: (p,α), (p,n), (p,p), (p,γ), (p,d) and others. The (p,α) reactions are usually exothermic. On heavy nuclei, their probability is not high, because the escape of an α particle from the nucleus is strongly hampered by a high Coulomb barrier, which allows only the fastest α particles to escape beyond the nucleus, the emission of which corresponds to the transition of the nucleus to the lower and, therefore, most sparsely located energy levels. And since the static weight of the state is determined by the density of levels, this implies a low probability of reactions of the type (p,α). This rule does not apply to light nuclei with a low Coulomb barrier.

The reactions (p,n) on stable nuclei are always endoenergetic and have a threshold whose value is greater than 0.8 MeV (usually 1 ÷ 3 MeV). Due to the fact that, during a (p,n) reaction, the product nucleus acquires an additional positive electric charge, it, as a rule, exhibits β^+^ or K-activity.

In the case when the kinetic energy of the incident protons exceeds the height of the Coulomb barrier, the probability of (p,p) reactions is comparable to the probability of reactions of the (p,n) type.

Since the probability of emission of particles by an intermediate nucleus is much higher than the probability of emission of a γ-quantum, a reaction of the (p,γ) type has a very low yield. However, in cases where the emission of particles is impossible or very difficult, the (p,γ) reaction becomes of great importance. For example, when E_p_ < E_min_ for a (p,n) reaction, a reaction of the (p,γ) type can go along with a (p,p) reaction.

The reactions (p,d) are much less common than others. Reactions of the type (p,d) are endothermic. In the case of the protons and heavier ions moving too slowly to overcome the Coulomb barrier when approaching the nucleus, it is necessary to create a relatively slowly varying electric field that acts on the protons of the nucleus. In these cases, the nucleus, absorbing electromagnetic energy, goes into an excited state, and the incident ion loses part of its energy [31].

So, in our work, irradiation is carried out using protons, and the (p,n) reaction is most probable.

The theoretical description of the calculation is detailed in the Supplementary Materials and Section 2. The initial stage of the calculation consists of analyzing the interaction curves of the elements that make up the perovskites. To do this, we use the database on nuclear reactions (EXFOR) of the Data Center for Photonuclear Experiments of Moscow State University (CDFYE MSU) [32]. We analyze the interaction curves presented in the experimental data and select the energy range that we need, namely from 1 to 24 MeV. Let us carry out these actions with all the elements included in the samples. As an example, please see the analysis for cesium (Supplementary Figure S19).

Based on the results of the analysis of the interaction curves of the EXFOR database, we draw up the interaction curves for each element included in the samples. When taking into account data, we focus on the year that the experiment was conducted and take the latest data. As a result, we obtain interaction curves in a suitable energy range. The obtained curves are presented in Figure 7.

For the excitation function of the ^206^Pb(p,n)^206^Bi reaction in the region from 10 to 12 MeV, we independently selected the values for constructing a straight line in this region. Since the interaction curve for lead does not have data in the region from 17 to 24 MeV, here we perform further calculations for the proton energy of 16 MeV to trace the contribution of each element to the final activity.

Let us calculate the specific ionization losses of a proton with an energy level of 16 MeV in the CsPbBr_3_ sample, using Formula (4): sample thickness, 300 nm; density, 4.57 g/cm^3^; the charge of nuclei in positron units is equal to 242 (the sum of the charge numbers of all elements of the sample composition); particle charge in positron units, 1; for an energy level of 16 MeV, the speed is 5.537 × 10^7^ m/s; mass number of nuclei of the medium substance, 579.817 (sum of mass numbers of elements of the sample composition). The final answer can be given in a more convenient version for us: dE/dx = 40.481 MeV/cm.

Let us calculate at what thickness a particle with an energy of 16 MeV will lose 1 MeV of its energy. For this, we use the formula ∆E = |dE/dx|∙∆x, where ∆E is the energy lost by the particle in the layer ∆x; knowing the lost energy and specific ionization losses of the proton, we can find the thickness of the layer (∆x = 0.025 cm). Since the value of the resulting layer thickness is much greater than the thickness of the sample itself, in activity calculations, we take into account only one energy value of 16 MeV and the value of the layer thickness as the total thickness of the sample. The calculations’ results are presented in Table 1. Then, we carry out similar calculations for the CsPbI_3_ sample: sample thickness, 300 nm; density, 4.84 g/cm^3^; the charge of nuclei in positron units is equal to 296; particle charge in positron units, 1; the speed is 5.537 × 10^7^ m/s; mass number of nuclei of the medium substance, 720.82.

To calculate the activation of the sample, we use Supplementary Equation (S3). Since the protons almost do not lose their initial energy when passing through a sample (the thickness of the sample is much less than the calculated thickness of the layer, when the proton will lose 1 MeV of its initial energy), we take into account only the interaction cross-section for an energy level of 16 MeV for all elements in the sample. The final activity is calculated as the sum of all activities of the elements multiplied by the coefficient of presence in the sample: Cs—0.2; Pb—0.2; and Br—0.6 (for the CsPbI_3_ sample, the coefficients will be equal to Cs—0.2; Pb—0.2; and I—0.6, respectively). Final activity values for perovskite samples: CsPbI_3_—2.0 kBq; and CsPbBr_3_—442.2 kBq. It is worth noting that the obtained values are relatively low. So, irradiation under the same conditions for highly acidic water (containing the ^18^O isotope) gives activity that is six orders of magnitude more. Thus, the irradiation of perovskites does not create significant induced activity, with the exception of short-lived isotopes formed by the interaction of protons with an organic cation. Consequently, the resulting decay products will have only a negligible effect on the formation of defects compared to the proton beam and displaced atoms.

### 3.3. Effect of Defects on the PCE and Electrophysics Characteristics of PSC

The second portion of the simulation is performed in Solar Cell Capacitance Simulator (SCAPS-1D) software, which was developed by the University of Gent, Belgium. SCAPS has several built-in spectrums for AM1.5G light. On the other hand, SCAPS provides the possibility to use a user-defined distribution of defects. The vacancy profile defined from TRIM calculations from the previous part with a different total defect density was used as the input data. It is worth noting that, since protons also create the significant defects in CTL, we changed the density of defects in them as well. From the results of SCAPS, the current density (J) vs. voltage (V) curve, the external quantum efficiency (EQE) curve, the band diagrams and the electron–hole pair generation–recombination curves were analyzed.

Given that we did not see the large numbers of defects being formed in perovskites by protons in the SRIM simulations, the calculations using SCAPS indicate that only very high defect densities have a significant impact on both the current–voltage characteristics and the EQE (Figure 8 and Figure 9).

**Figure 2:**
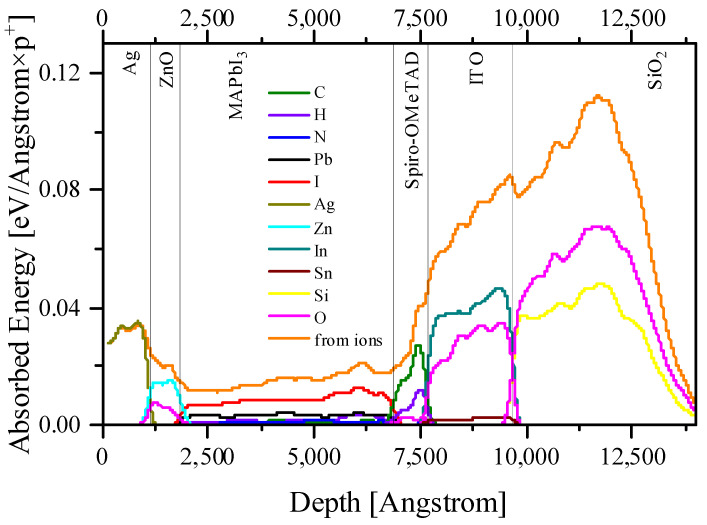
The total energy absorbed by target (solar cell) atoms from a 0.15 MeV proton beam.

**Figure 3:**
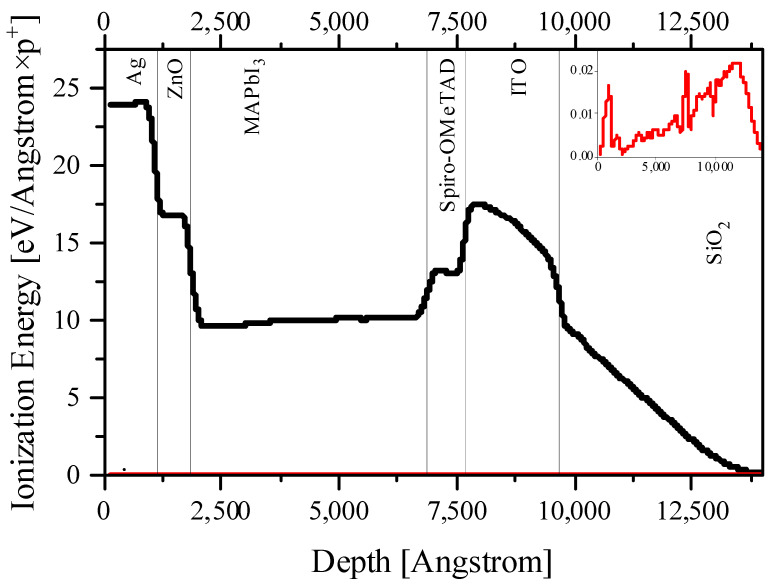
The energy spent on ionization by displaced atoms and protons (on the insert) for the entire solar cell architecture for 0.15 MeV protons.

**Figure 4:**
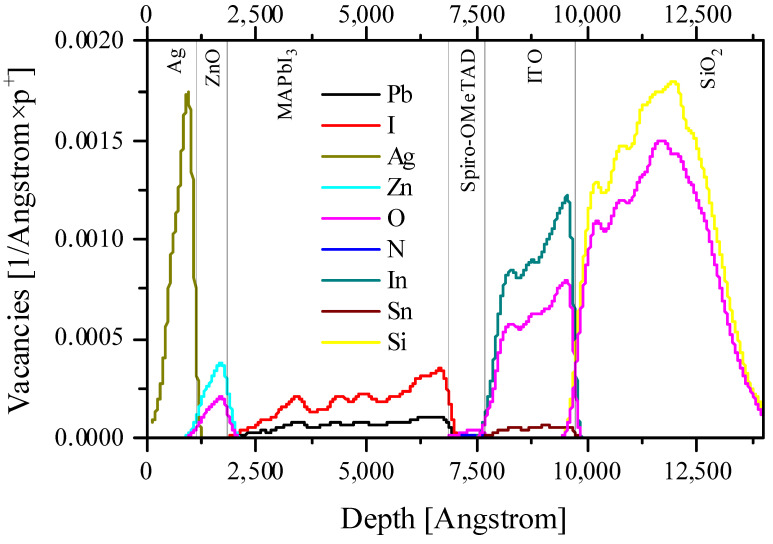
The energy spent on the formation of vacancies by protons for the entire solar cell architecture for 0.15 MeV protons.

**Figure 5:**
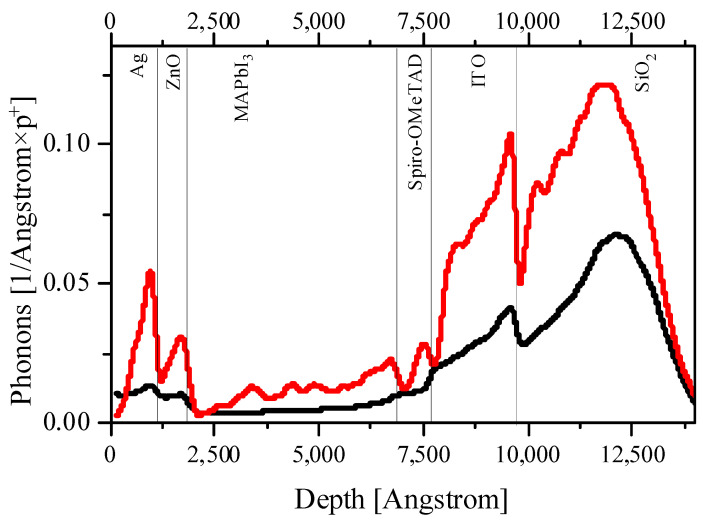
The energy spent on the formation of phonons by protons and displaced atoms for the entire solar cell architecture for 0.15 MeV protons. Red line, recoil atoms; black line, protons.

**Figure 6:**
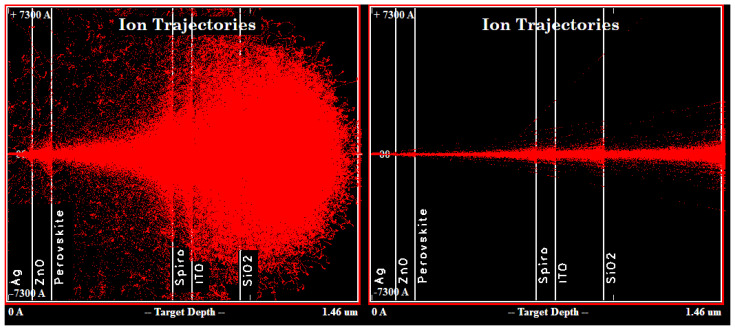
Calculation of the range for protons with energies of 0.15 MeV (**left** panel) and 3 MeV (**right** panel) in the CsPbI_3_ sample.

**Figure 7:**
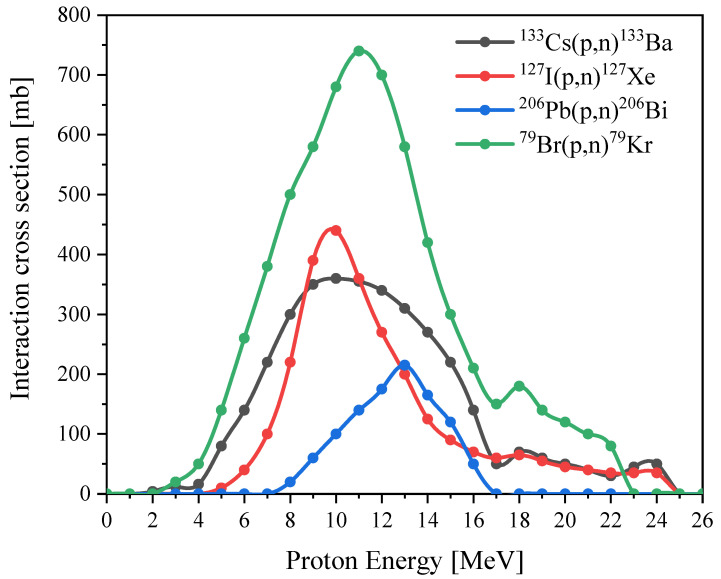
The reaction excitation function for ^133^Cs(p,n)^133^Ba, ^127^I(p,n)^127^Xe, ^206^Pb(p,n)^206^Bi and ^79^Br(p,n)^79^Kr.

**Figure 8:**
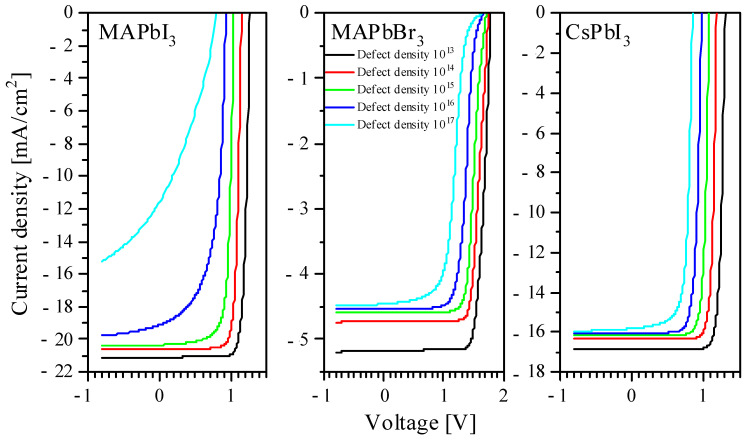
The current–voltage characteristics of solar batteries at different defect densities.

**Figure 9:**
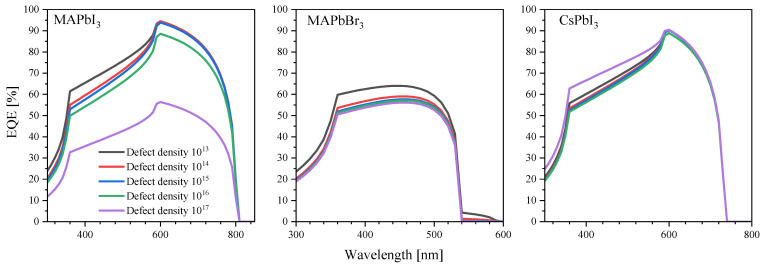
External quantum efficiency at different defect densities.

Taking into account that exposure to protons has a much more significant effect on the defect formation in CTL (Figure 2, Figure 3, Figure 4 and Figure 5), it would be logical to expect that the degradation of the current–voltage characteristic and the drop-in quantum efficiency is associated precisely with the weakening of the conductivity of the CTL and the removal of charges from the place of their origin and the increase recombination rates at the resulting defects. However, the band diagrams and the electron–hole pair generation–recombination curves presented in Supplementary Figures S20–S24 do not support these findings. Even the largest defect densities specified in our simulations only result in a change in the Fermi level for minority charge carriers in the CTL (Supplementary Figure S20). In addition, a change in the density of minority charge carriers in the CTL due to the appearance of defects is also observed (Supplementary Figure S21). However, defects in the CTL do not have a significant effect on the current density minority charge carriers in the CTL (Supplementary Figure S22), generation (Supplementary Figure S23) and nonradiative recombination of charge carriers at defects (Supplementary Figure S24). We also note that the current density of the majority of charge carriers in the CTL decreases with the increasing defect density, which is logically associated with an increase in the scattering of charge carriers by defects. However, defects do not capture charge carriers in the CTL, which is reflected in the absence of changes in nonradiative recombination in the CTL. In this case, defects do not affect the density of carriers, the creation of which occurs in the perovskite in interband transitions.

On the other hand, the appearance of defects in perovskite films significantly affects the current densities and Shockley–Read–Hall recombination (Supplementary Figure S24) and current density. Thus, despite the low probability of defects occurring in the perovskite layer compared to CTL, the appearance of defects in perovskite film will significantly affect the nonradiative recombination of electron–hole pairs on it. Thus, it was previously shown that an increase in the density of defects in the middle of the gap to 10^15^ cm^−3^ negatively affects the performance of the device [33]. Therefore, proper protection of the perovskite layer by the substrate, electrodes and CTL is important, especially given the minimal impact of defects in the CTL on the electrical parameters of the solar cell.

At the same time, we note that, at doses acquired by a solar cell over several years of operation in orbit, there is no noticeable drop in the efficiency. Thus, only the defects accumulated at very high doses will be significant, which does not play a significant role in space applications, since their accumulation rate is comparable to the lifetime of space craft.

## 4. Conclusions

To date, the main studies on the effect of cosmic radiation on the stability of perovskite solar cells have been undertaken for electrons, X-rays and gamma rays. This study fills, to a certain extent, the gap associated with proton exposure. For terrestrial tests, the most indicative today is the impact of proton beams with an energy of 0.05–0.15 MeV. We clearly show that such energy can only work for perovskite in isolation from the actual solar panel architecture. For the terrestrial testing of solar cells, the most relevant energy would be several MeV. We made an attempt to carry out the first systematic study of the influence of perovskite composition on the stability of the characteristics of solar panels under the influence of proton beams. Due to certain difficulties in carrying out the experimental measurements at the first stage, we performed the well-tested SRIM and SCAPS calculations for proton energies of 0.15, 3.0 and 18 MeV. Furthermore, we present, for the first time, the calculation of the radiation-induced activation effect in hybrid perovskites. The obtained data confirm the previous results from the gamma and electron stability studies, which show that FAPbI_3_ perovskite has a great potential for use in space [30]. On the other hand, we have shown the importance of taking into account the lateral distribution of the incident proton beam throughout the entire depth of the solar cell. It turned out that most protons at any energy give up a significant part of their energy to charge transfer layers (CTLs) and electrodes. It was shown that, for protons with an energy of several MeV, the release of their energy can be considered uniform over the depth and area of the entire solar cell, and fairly simple protection against the low-energy protons can be used, for example, in the form of transparent plastic films. This leads us to the conclusion that it is necessary to conduct the terrestrial tests with protons of several MeV energies. The studies carried out, on the one hand, made it possible to numerically evaluate the effect of protons of various energies in the MeV range on APbX_3_ perovskites, and on the other hand, to select the most radiation-resistant compositions of these materials. Thus, the results obtained are both directly predictive for the design of solar cells in space photovoltaic devices and create a theoretical basis for subsequent terrestrial experiments.

## Figures and Tables

**Table 1:** Calculation of energy loss by a proton in a perovskite sample.

Sample	Proton Energy, MeV	v, m/s	β	dE/dx, MeV/cm	∆x, cm
CsPbBr_3_	16	5.537·10^7^	0.185	40.481	0.025
CsPbI_3_	16	5.537·10^7^	0.185	38.539	0.026

## Data Availability

The data are contained within the article.

## Supplementary Materials

The following supporting information can be downloaded at https://www.mdpi.com/article/10.3390/nano14010001/s1, Figure S1: A heavy nonrelativistic charged particle with charge (ze) and speed (v) flies along the x-axis at a distance (b) from the electron; Figure S2: The energy transferred to the displacement of target atoms at a proton beam energy of 0.15 MeV; Figure S3: The energy spent on ionization of target atoms by displaced atoms at a proton beam energy of 0.15 MeV; Figure S4: The energy spent on the formation of vacancies at a proton beam energy of 0.15 MeV; Figure S5: The energy spent on the formation of phonons by protons and displaced atoms at a proton beam energy of 0.15 MeV; Figure S6: The energy transferred to the displacement of target atoms at a proton beam energy of 3 MeV; Figure S7: The energy spent on the ionization of target atoms by displaced atoms at a proton beam energy of 3 MeV; Figure S8: The energy spent on the formation of vacancies at a proton beam energy of 3 MeV; Figure S9: The energy spent on the formation of phonons by protons and displaced atoms at a proton beam energy of 3 MeV; Figure S10: The energy transferred to the displacement of target atoms at a proton beam energy of 18 MeV; Figure S11: The energy spent on ionization of target atoms by displaced atoms at a proton beam energy of 18 MeV; Figure S12: The energy spent on the formation of vacancies at a proton beam energy of 18 MeV; Figure S13: The energy spent on the formation of phonons by protons and displaced atoms at a proton beam energy of 18 MeV; Figure S14: Comparison of the energy transferred to the displacement of target atoms for perovskites with a different A-cation at a proton beam energy of 0.15 MeV; Figure S15: Comparison of the energy spent on the formation of vacancies for perovskites with a different A-cation at a proton beam energy of 0.15 MeV; Figure S16: Calculation of the range for protons with energies of 0.15 MeV; Figure S17: Calculation of the range for protons with energies of 3 MeV; Figure S18: Calculation of the range for protons with energies of 18 MeV; Figure S19: Formation of 128-, 131-Ba and 132-Cs in reactions induced by protons on the Cs target, and 127- and 129-Cs in reactions induced by 3,4-He on the I target; Figure S20: Influence of defect density on band structure of PSC; Figure S21: Influence of defect density on carrier density of PSC; Figure S22: Influence of defect density on current density of PSC; Figure S23: Influence of defect density on electron–hole generation function of PSC; Figure S24: Influence of defect density on Shockley–Read–Hall recombination function of PSC; Table S1: Initial material parameters used for charge transport layers; Table S2: Initial perovskite material parameters.
