# Supplementary material for: Proton Irradiation on Halide Perovskites: Numerical Calculations
Source: Nanomaterials (Basel). 2023 Dec 19;14(1):1. doi: 10.3390/nano14010001 (PMC10780994; doi:10.3390/nano14010001)
Supplement: Supplementary file 1 [file nanomaterials-14-00001-s001.zip › nanomaterials-2762625-supplementary.pdf]

Supporting Information

Proton Irradiation on Halide Perovskites: Numerical Calculations

Alexandra V. Rasmetyeva [1], Stepan S. Zyryanov [1], Ivan E. Novoselov [1], Andrey I. Kukharenko [1,2], Efrem V. Makarov [3], Seif O. Cholakh [1], Ernst Z. Kurmaev [1,2] and Ivan S. Zhidkov [1,2,*]

[1] Institute of Physics and Technology, Ural Federal University, Mira 19 Street, Yekaterinburg 620002, Russia
[2] M.N. Mikheev Institute of Metal Physics of Ural Branch of Russian Academy of Sciences, S. Kovalevskoi 18 Street, Yekaterinburg 620108, Russia
[3] Institute of Electrophysics of Ural Branch of Russian Academy of Sciences, Amundsena 106 Street, Yekaterinburg 620110, Russia
* Correspondence: i.s.zhidkov@urfu.ru

Theoretical basis for determining activity

The activation is the process of producing radioactive nuclei from the stable nuclei. In terms of their physical nature, the artificial radioactive nuclei are no different from the natural ones, since the properties of the nuclei of a given radioactive nuclide do not depend on the method of its formation.

The activation cross section is a quantity that shows the probability of the formation of radioactive isotopes during the interaction of nuclear particles with atomic nuclei. It is expressed in cm^2 or barns (1 b = 10^{-24} cm^2), that is, it has the dimension of the surface into which the bombarding particle must hit. This value determines the probability of a nuclear reaction occurring. The cross section for reactions of a certain type strongly depends on the energy of the bombarding particles, i.e. the probability of a particular type of reaction occurring is a function of the energy possessed by the bombarding particle (in the form of kinetic energy).

If a radioactive nuclide is formed as a result of the nuclear reaction *C*(*x*,*y*)*D*, then it is necessary to take into account its decay during irradiation:

$$\frac{dN_D}{dt} = \sigma\Phi N_C - \lambda N_D. \quad \text{(S1)}$$

Carrying out the integration within the limits of $t = 0$ and $t =$ t (t is the irradiation time) and taking $N_D = 0$ at $t = 0$, we obtain the number of nuclei formed as a result of proton irradiation:

$$N_D(t) = \frac{\sigma\Phi N_C}{\lambda}\left(1 - e^{-\lambda t}\right), \quad \text{(S2)}$$

where Φ is the proton flux, i.e. the number of protons passing through 1 cm of the target in 1 second; N_C is the number of atoms of the activated isotope in the target; λ is the decay constant of the resulting isotope; *t* – irradiation time; σ – activation cross section.

The activity of a substance irradiated during time t, after time t* after the end of irradiation, is expressed by the formula [28]:

$$A(t, t^*) = \sigma \Phi N_C \left(1 - e^{-\lambda t}\right) \cdot e^{-\lambda t^*}. \quad \text{(S3)}$$

Theoretical basis for interaction of protons with matter

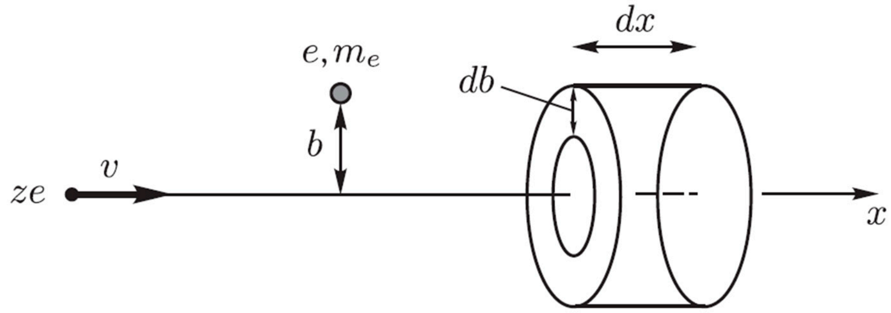

Figure S1. A heavy non-relativistic charged particle with charge ze and speed v flies along the x axis at a distance b from the electron.

The proton flux is equal to the number of protons passing through one square centimeter of the target per second, that is:

$$\Phi = \frac{I}{S} = \frac{q_p N_p}{tS} = \frac{N_p}{tS} = \frac{I}{q_p tS}, \quad \text{(S4)}$$

where I is the proton beam current; q_p – proton charge; N_p – number of protons; t – time (in our case equal to 1 second); S – target area (in our case equal to 1 cm^2).

Since it needed exactly the number of protons, one divides the current of the proton beam by the charge of one proton. It can be found that the initial number of particles in the target from the following formula:

$$N = \frac{N_A \rho V}{\mu} = \frac{N_A \rho S d}{\mu}, \quad \text{(S5)}$$

where N_A is Avogadro's number; ρ – sample density; V – sample volume; μ is the molar mass of the sample.

SRIM modeling results

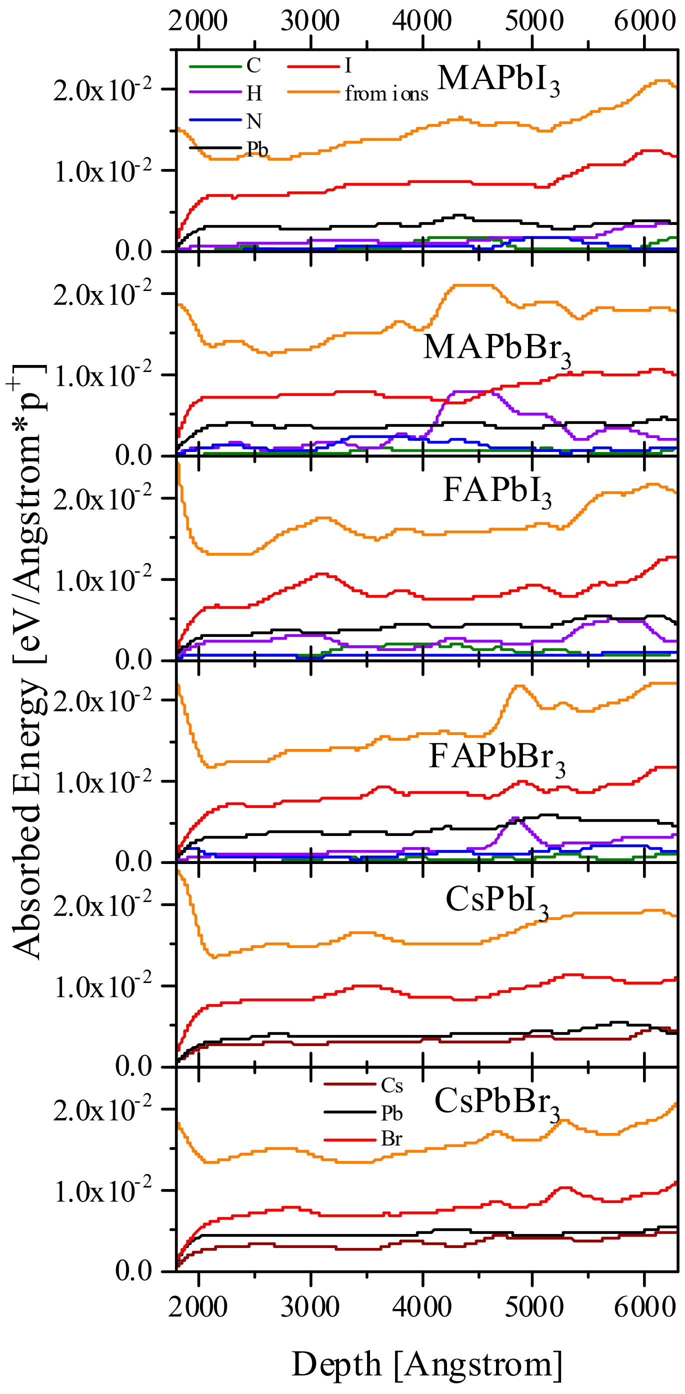

Figure S2. The energy transferred to the displacement of target atoms at a proton beam energy of 0.15 MeV.

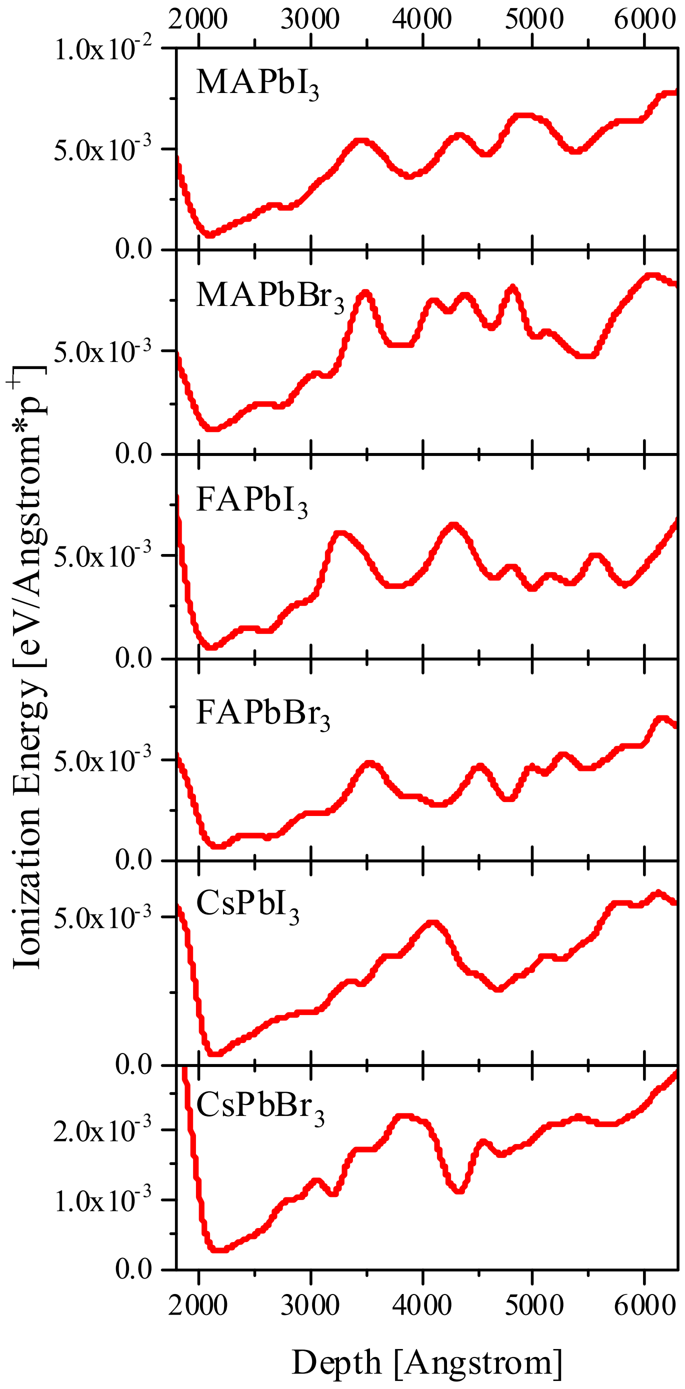

Figure S3. The energy spent on ionization of target atoms by displaced atoms at a proton beam energy of 0.15 MeV.

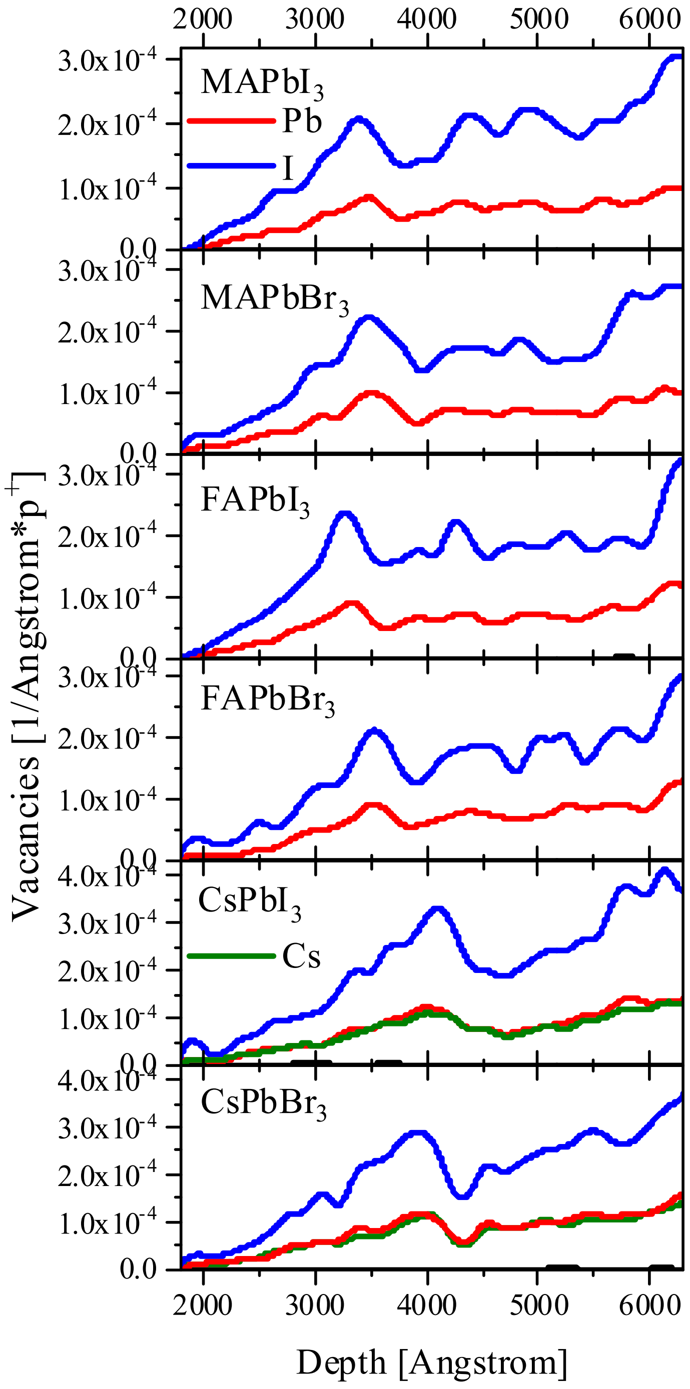

Figure S4. The energy spent on the formation of vacancies at a proton beam energy of 0.15 MeV.

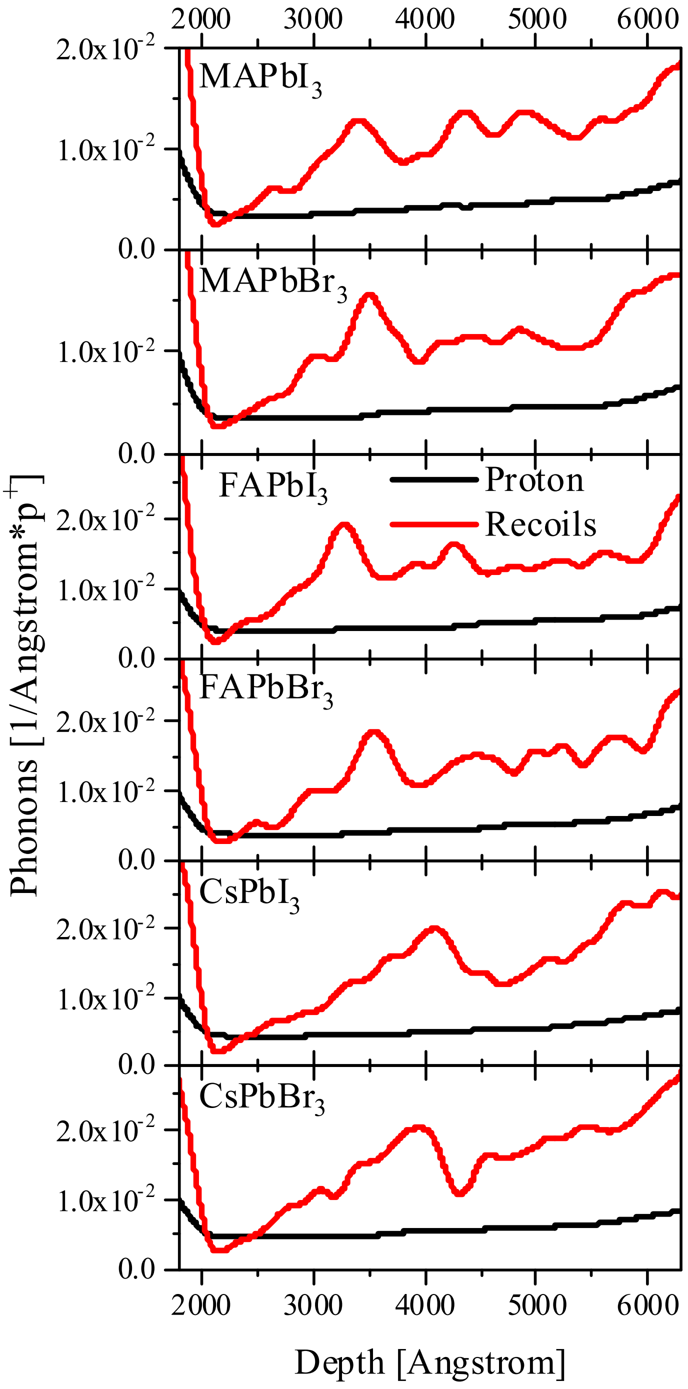

Figure S5. The energy spent on the formation of phonons by protons and displaced atoms at a proton beam energy of 0.15 MeV.

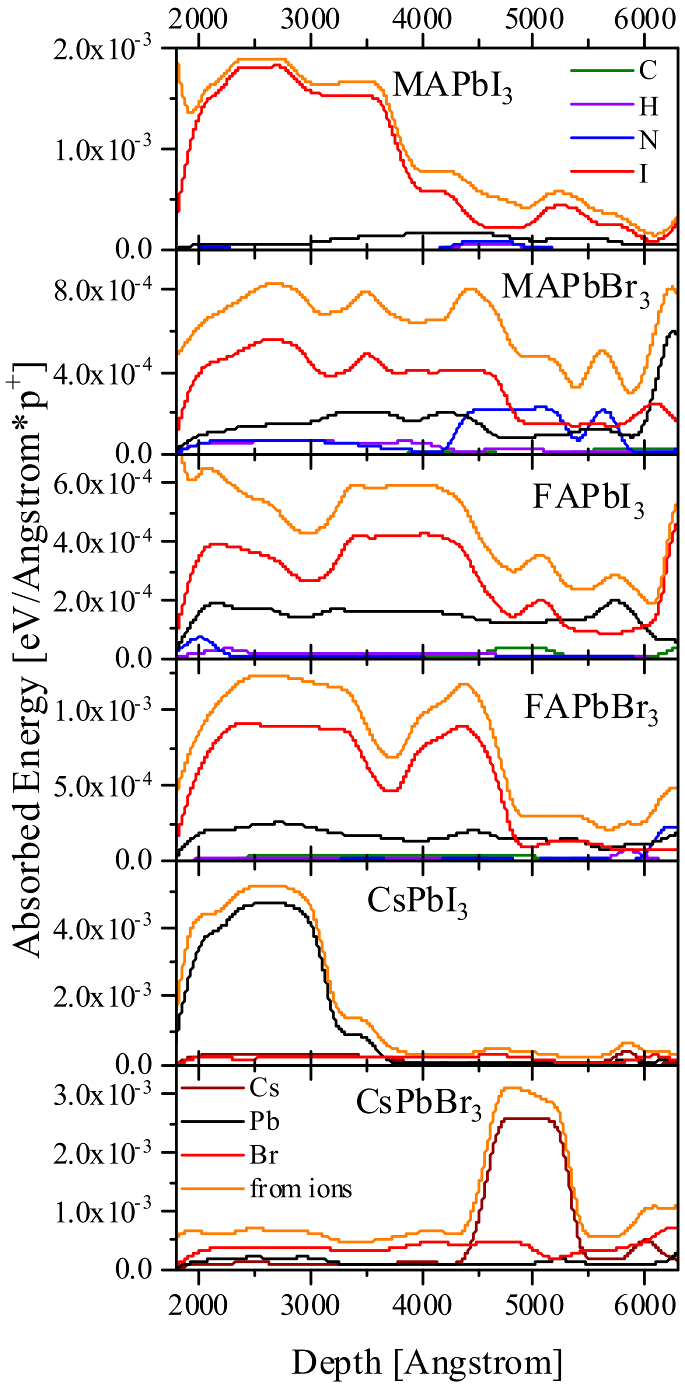

Figure S6. The energy transferred to the displacement of target atoms at a proton beam energy of 3 MeV.

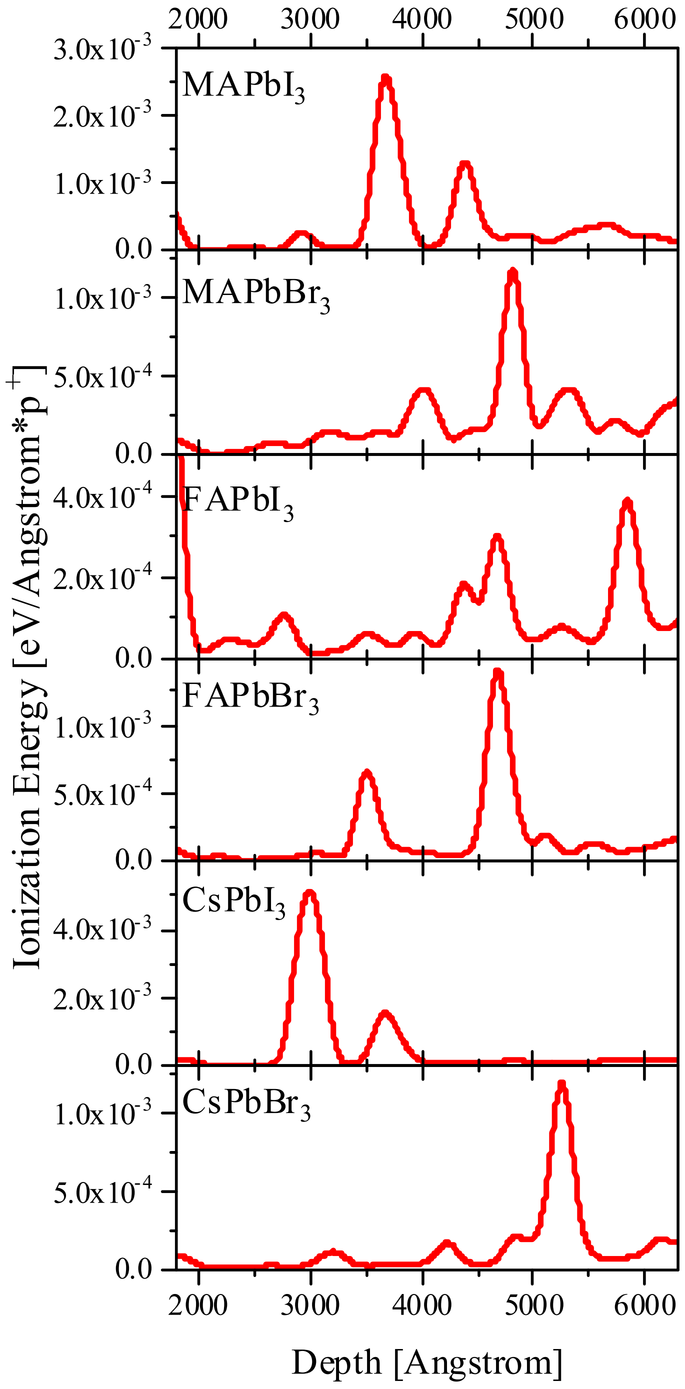

Figure S7. The energy spent on ionization of target atoms by displaced atoms at a proton beam energy of 3 MeV.

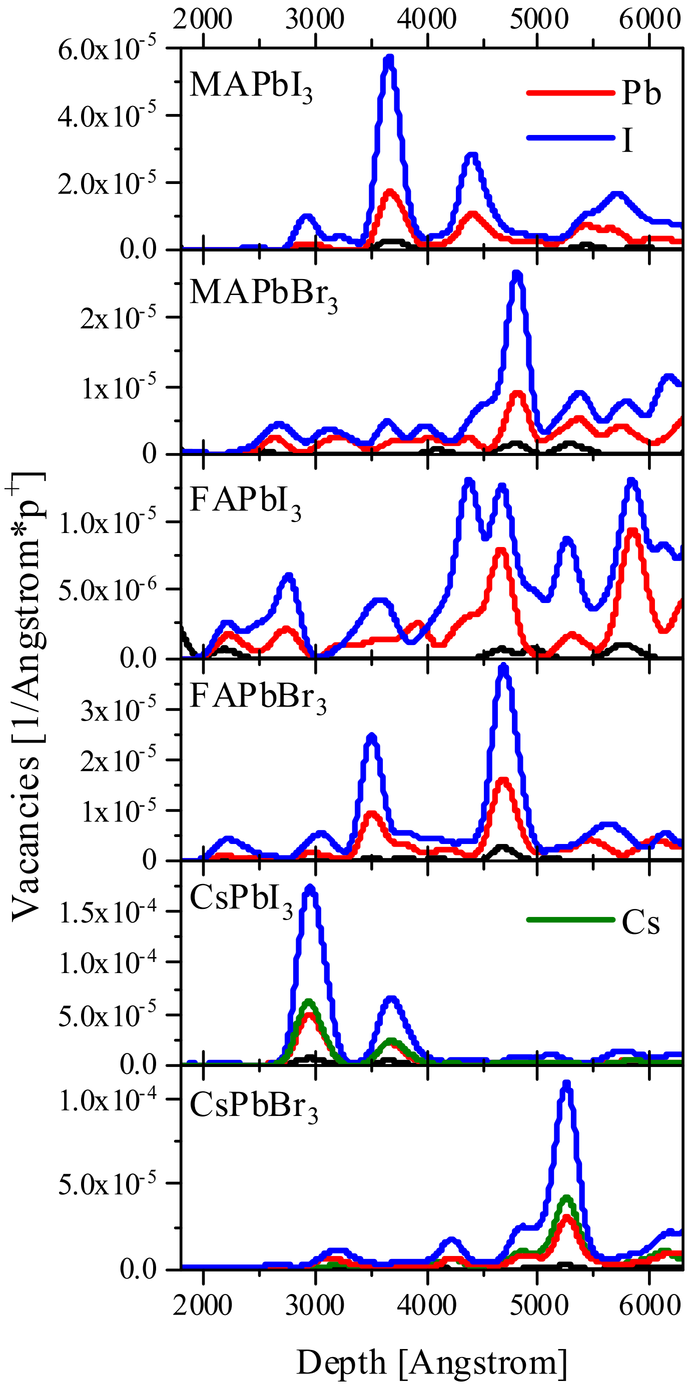

Figure S8. The energy spent on the formation of vacancies at a proton beam energy of 3 MeV.

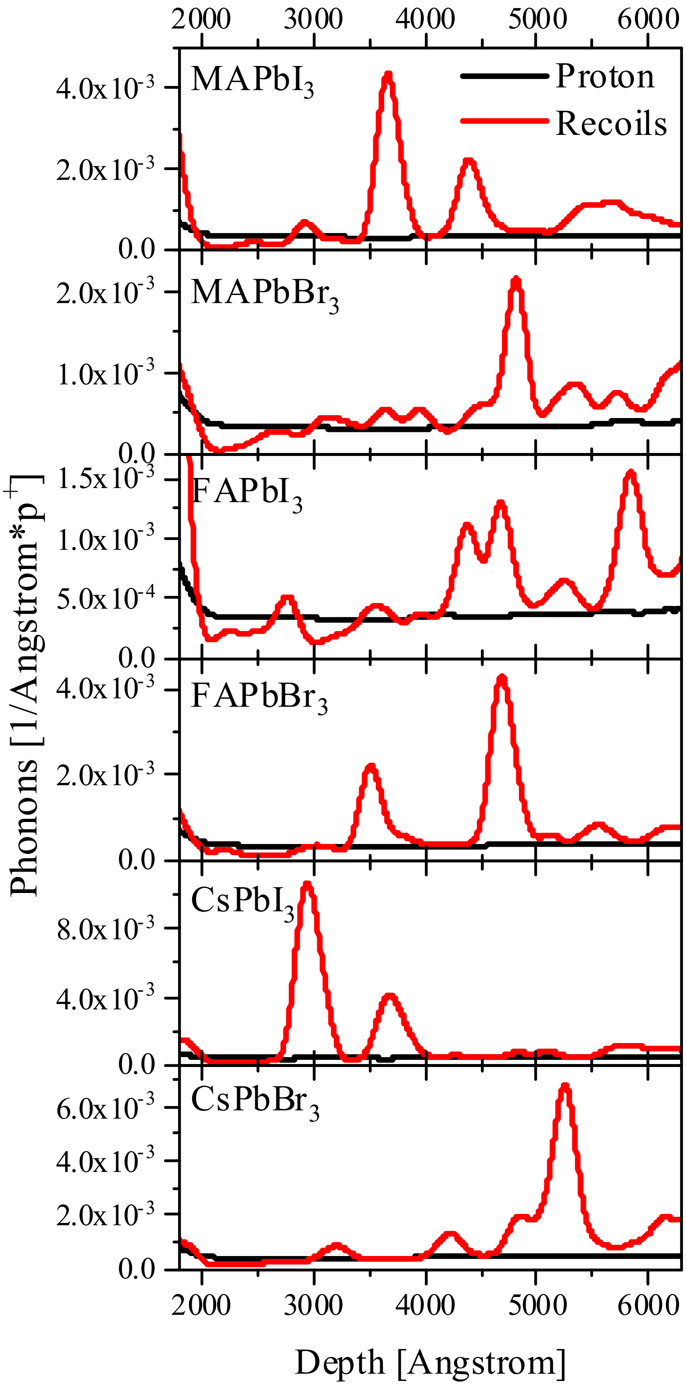

Figure S9. The energy spent on the formation of phonons by protons and displaced atoms at a proton beam energy of 3 MeV.

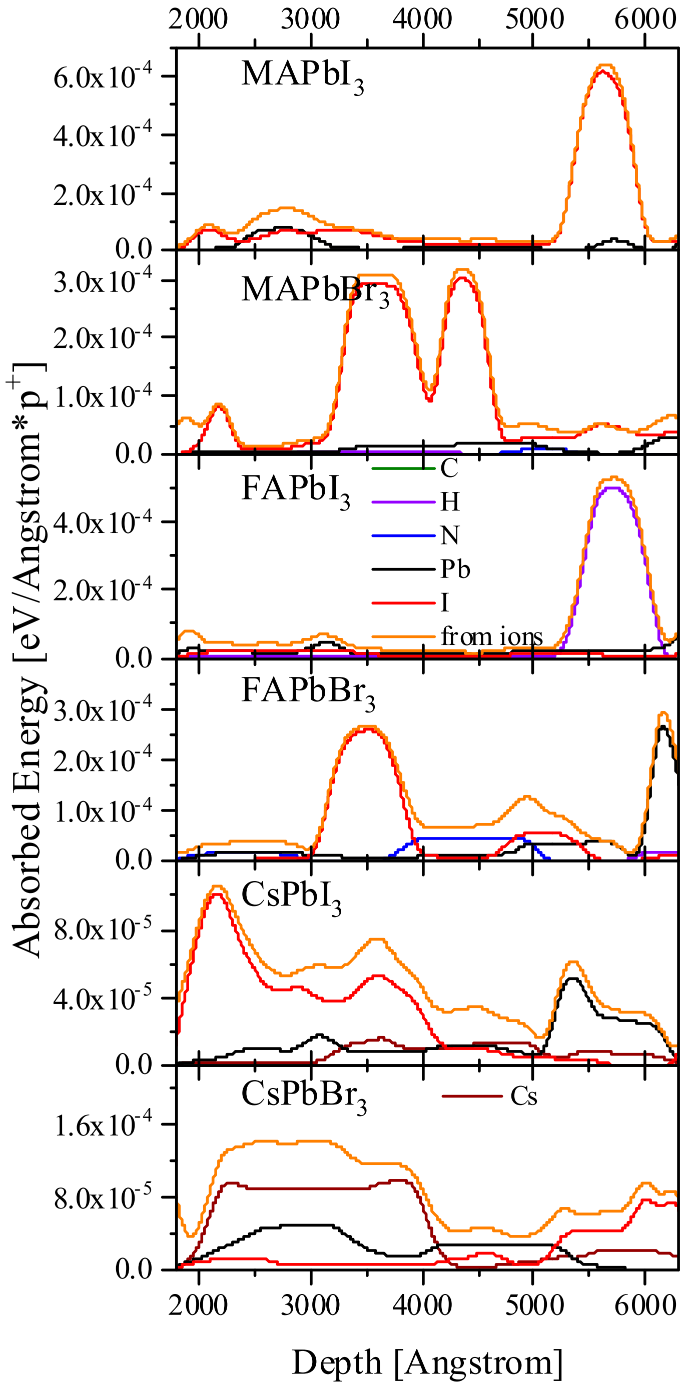

Figure S10. The energy transferred to the displacement of target atoms at a proton beam energy of 18 MeV.

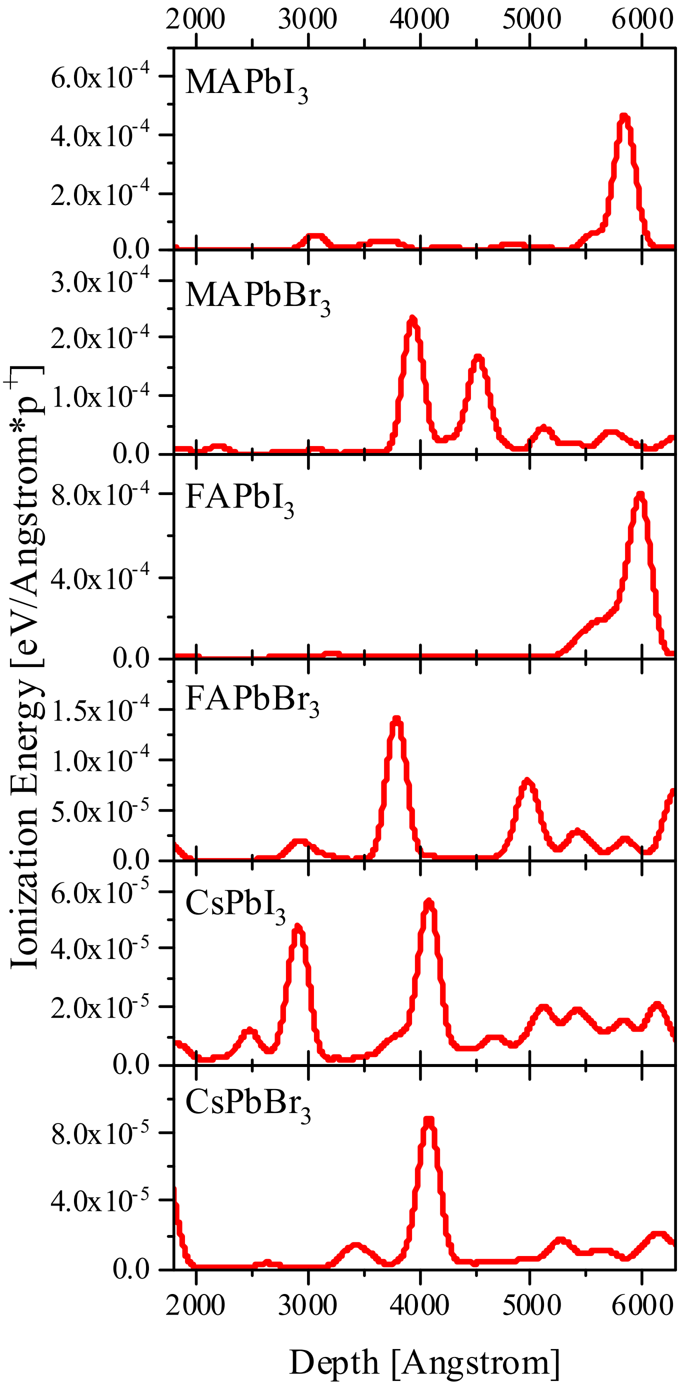

Figure S11. The energy spent on ionization of target atoms by displaced atoms at a proton beam energy of 18 MeV.

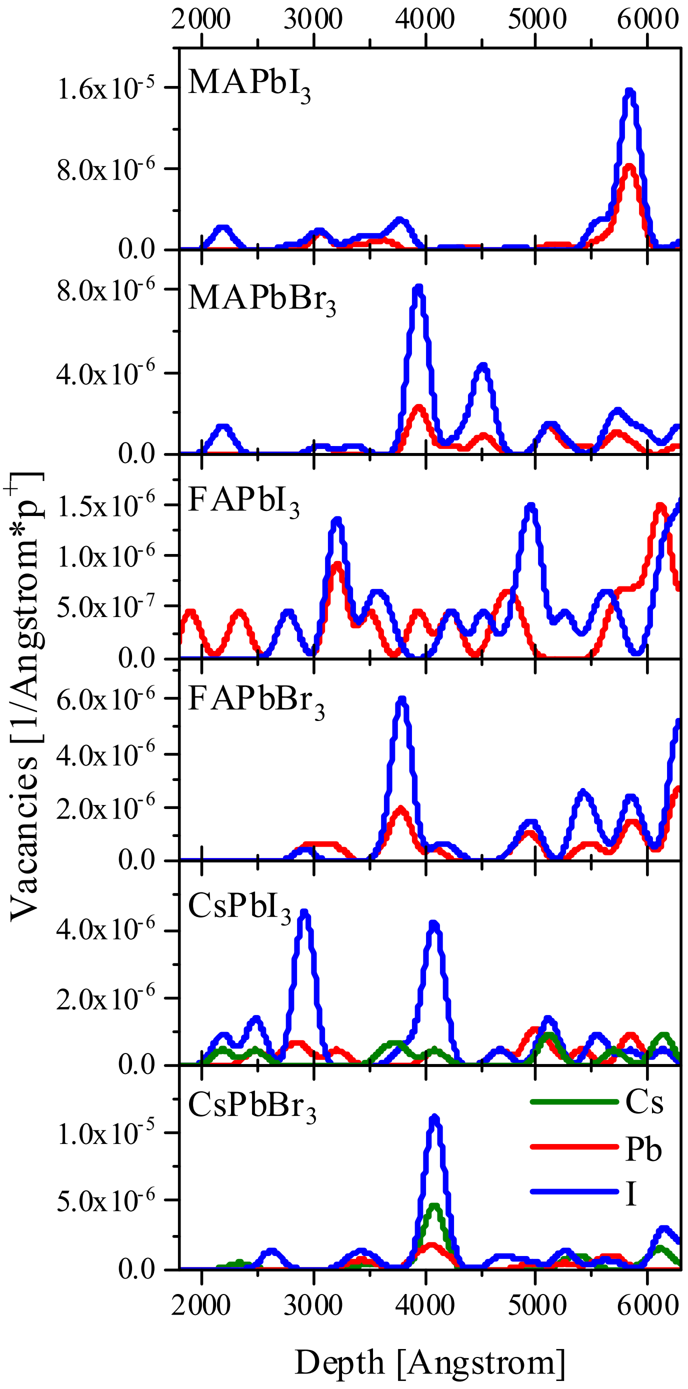

Figure S12. The energy spent on the formation of vacancies at a proton beam energy of 18 MeV.

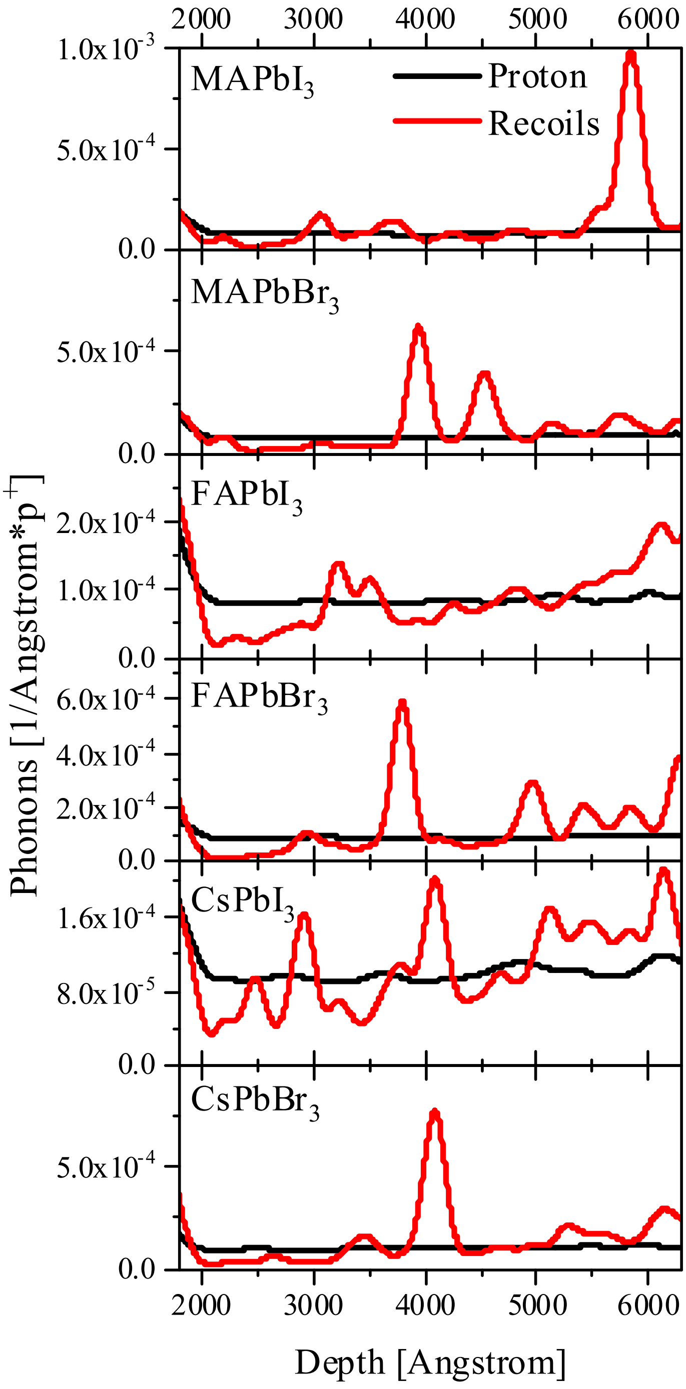

Figure S13. The energy spent on the formation of phonons by protons and displaced atoms at a proton beam energy of 18 MeV.

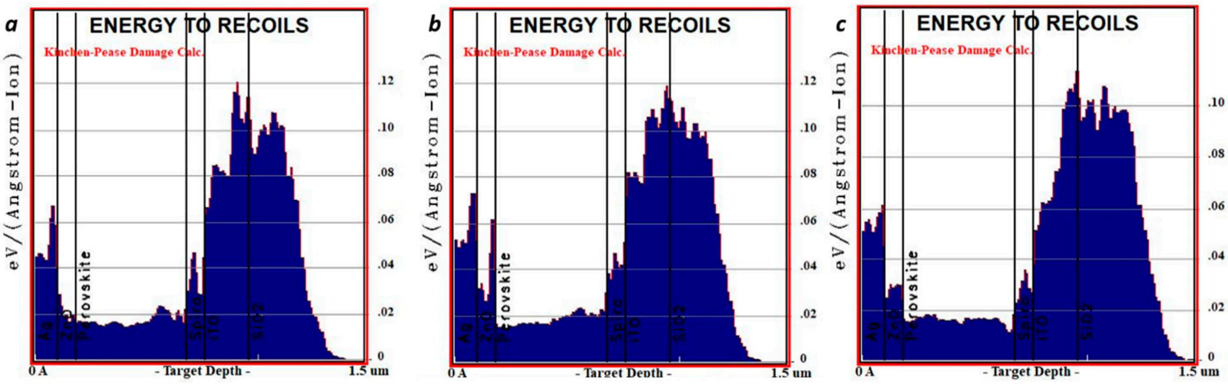

Figure S14. Comparison of the energy transferred to the displacement of target atoms for perovskites with a different A-cation at a proton beam energy of 0.15 MeV: a – $MAPbI_3$, b – $FAPbI_3$, c – $Cs_{0.12}FA_{0.88}PbI_3$.

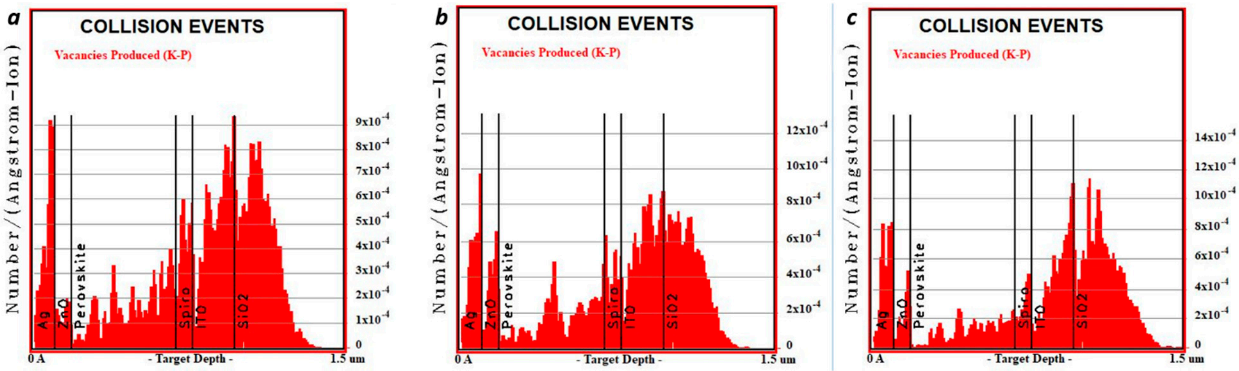

Figure S15. Comparison of the energy spent on the formation of vacancies for perovskites with a different A-cation at a proton beam energy of 0.15 MeV: a – $MAPbI_3$, b – $FAPbI_3$, c – $Cs_{0.12}FA_{0.88}PbI_3$.

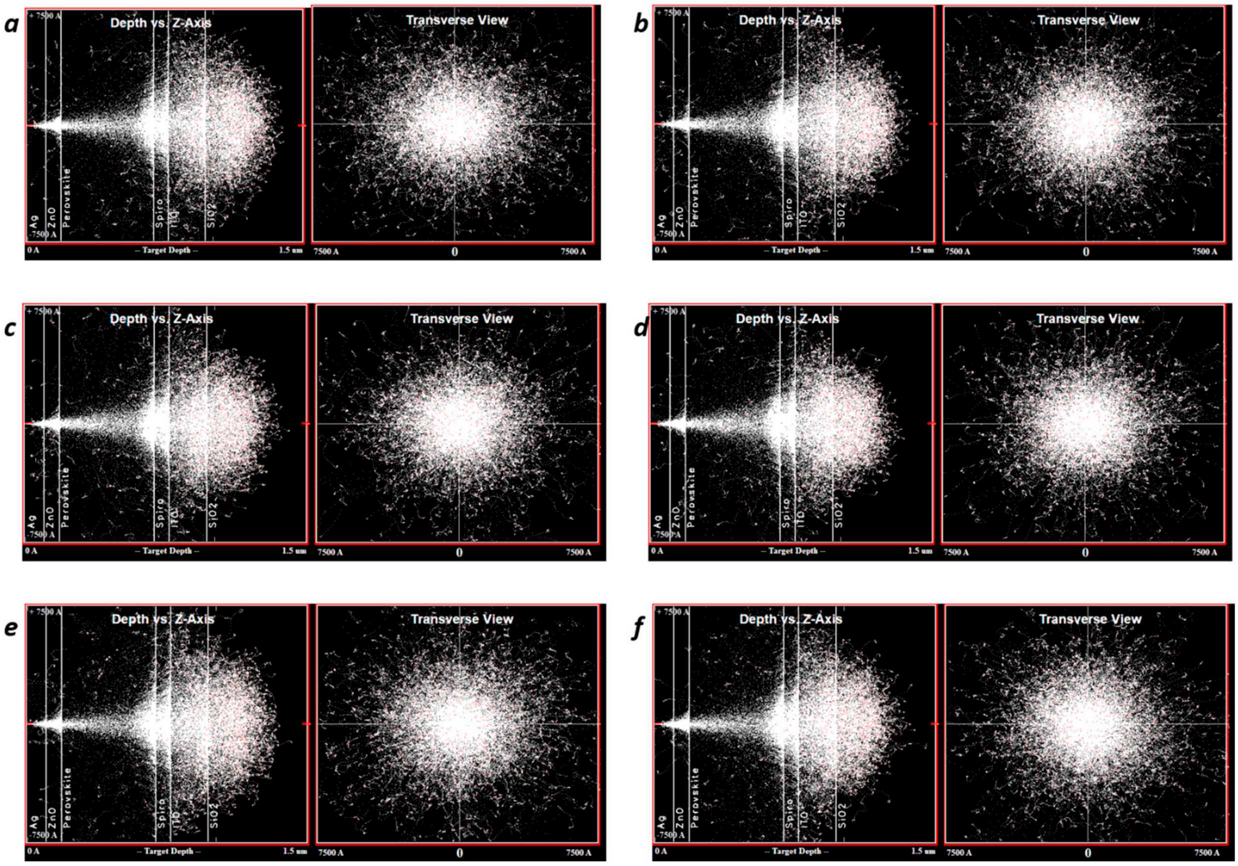

Figure S16. Calculation of the range for protons with energies of 0.15 MeV: a – $MAPbI_3$, b – $FAPbI_3$, c – $MAPbBr_3$, d – $FAPbBr_3$, e – $CsPbI_3$, f – $CsPbBr_3$ samples.

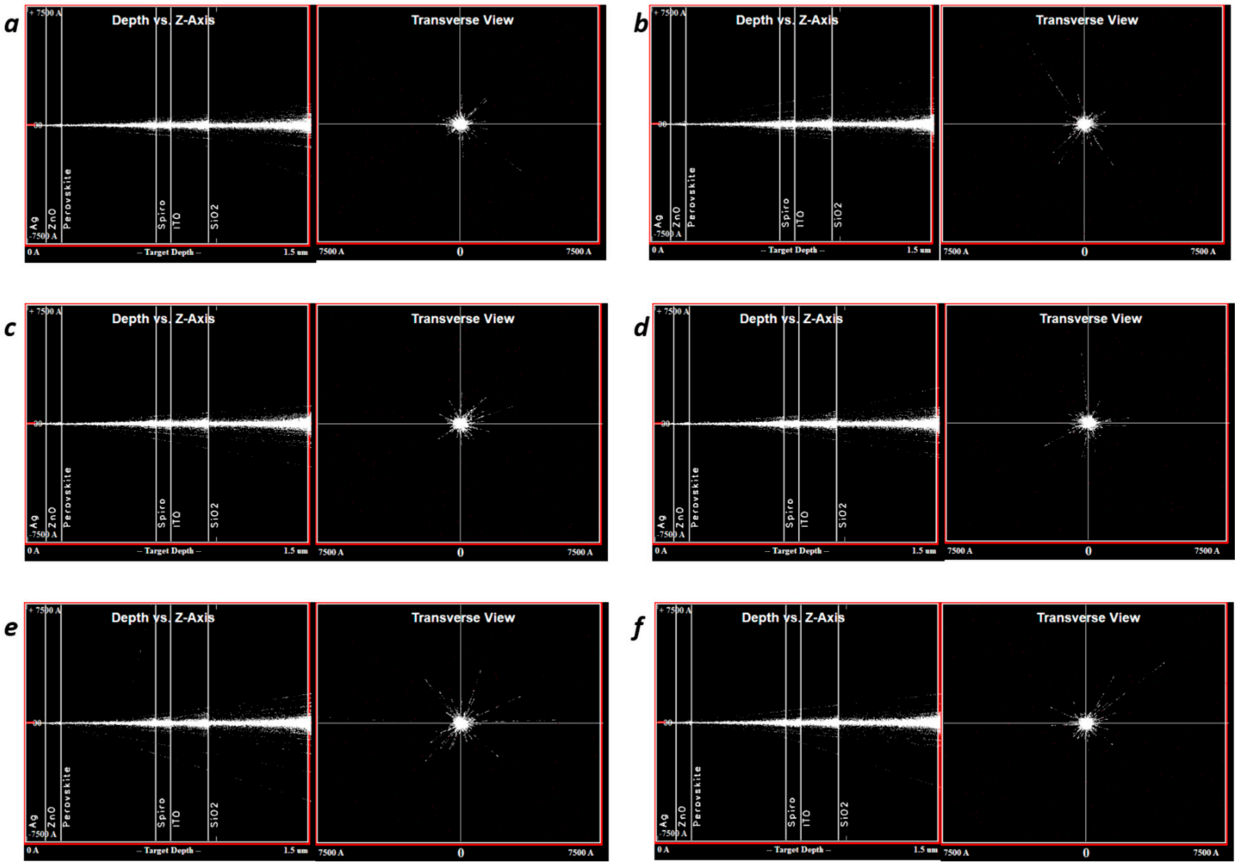

Figure S17. Calculation of the range for protons with energies of 3 MeV: a – $MAPbI_3$, b – $FAPbI_3$, c – $MAPbBr_3$, d – $FAPbBr_3$, e – $CsPbI_3$, f – $CsPbBr_3$ samples.

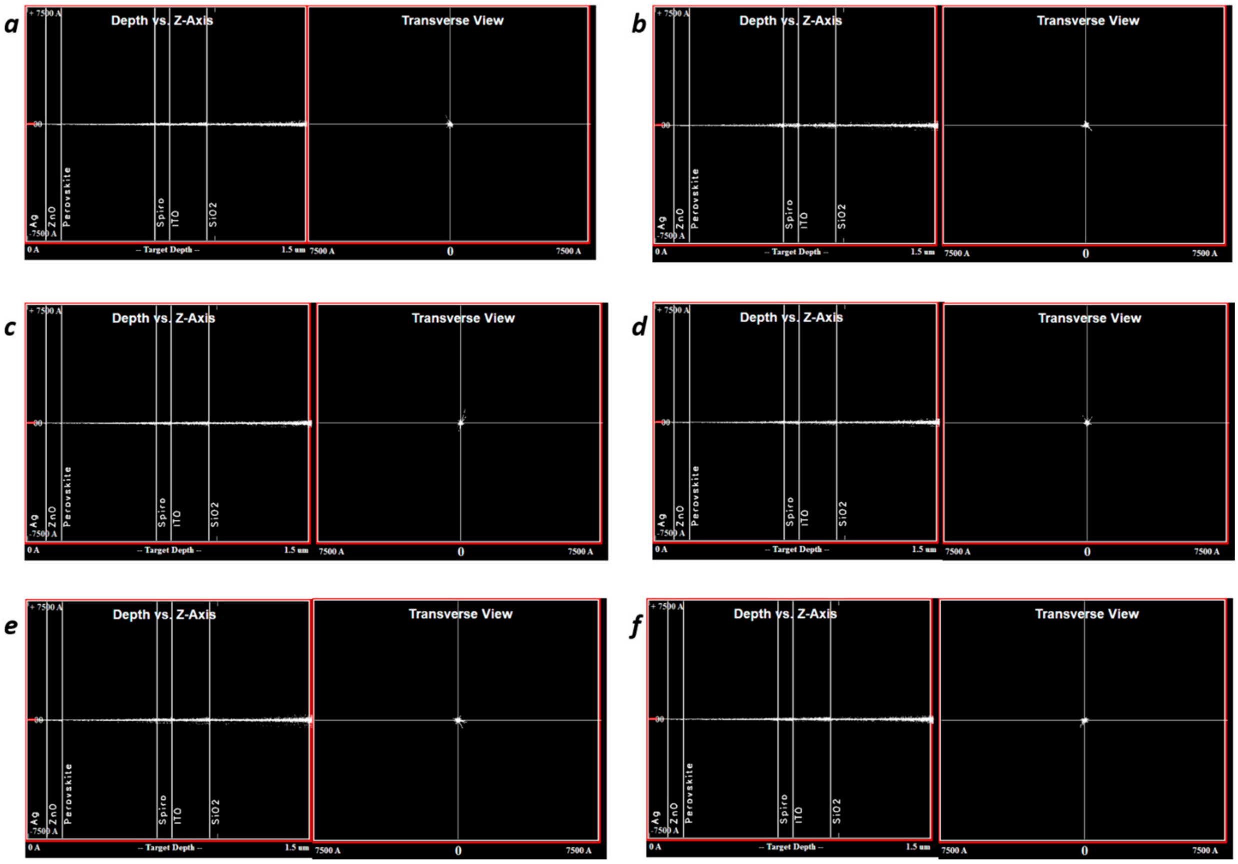

Figure S18. Calculation of the range for protons with energies of 18 MeV: a – $MAPbI_3$, b – $FAPbI_3$, c – $MAPbBr_3$, d – $FAPbBr_3$, e – $CsPbI_3$, f – $CsPbBr_3$ samples.

SUBENT	A0572004 20170205
AUTHOR	(C.Deptula,Kim Sen Han,O.Knotek,S.Mikolajewski, L.M.Popinenkova,E.Rurarz,N.G.Zaitseva)
TITLE	Production of 128,131-Ba,132-Cs in proton induced reactions on a Cs target and of 127,129-Cs in 3,4-He induced reactions on I target.
REFERENCE	(J,NKA,35,63,1990)
INSTITUTE	(3POLIPJ).Radioisotope Research and Development Center, Swierk,Poland. (3KDRPYN).Institute of Radiochemistry,Pyongyang,D.P.R. Korea. (3CZRUJV,4RUSFVE,4ZZZDUB,3POLIPJ)
INC-SOURCE	
FACILITY	(LINAC,4RUSFVE).Lu-100 at the Institute For High Energy Physics in Serpukhuv (RUSSIA).
REACTION	(55-CS-133(P,X)55-CS-132,,SIG) Independent

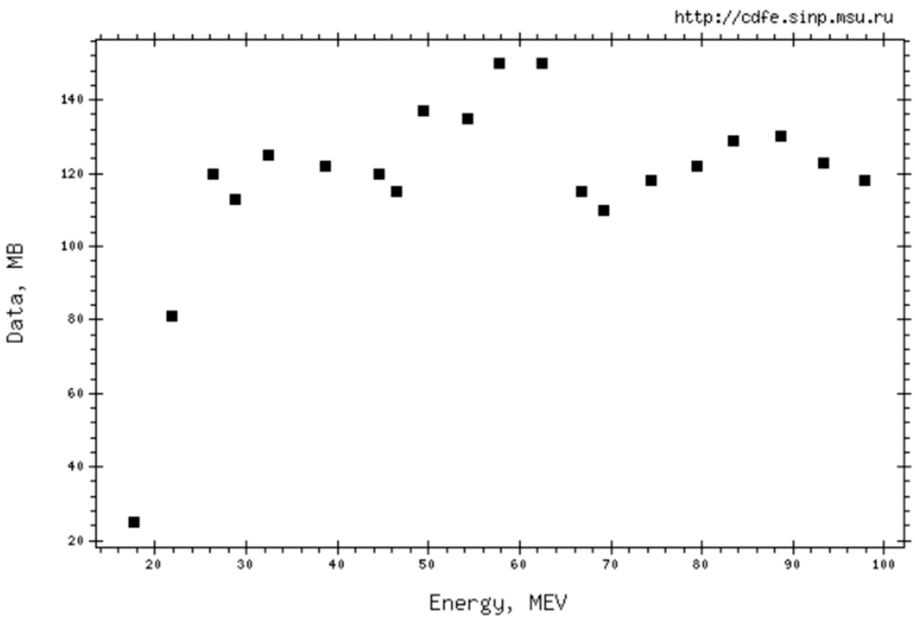

Figure S19. Formation of 128-, 131-Ba, 132-Cs in reactions induced by protons on the Cs target, and 127-, 129-Cs in reactions induced by 3,4-He on the I target

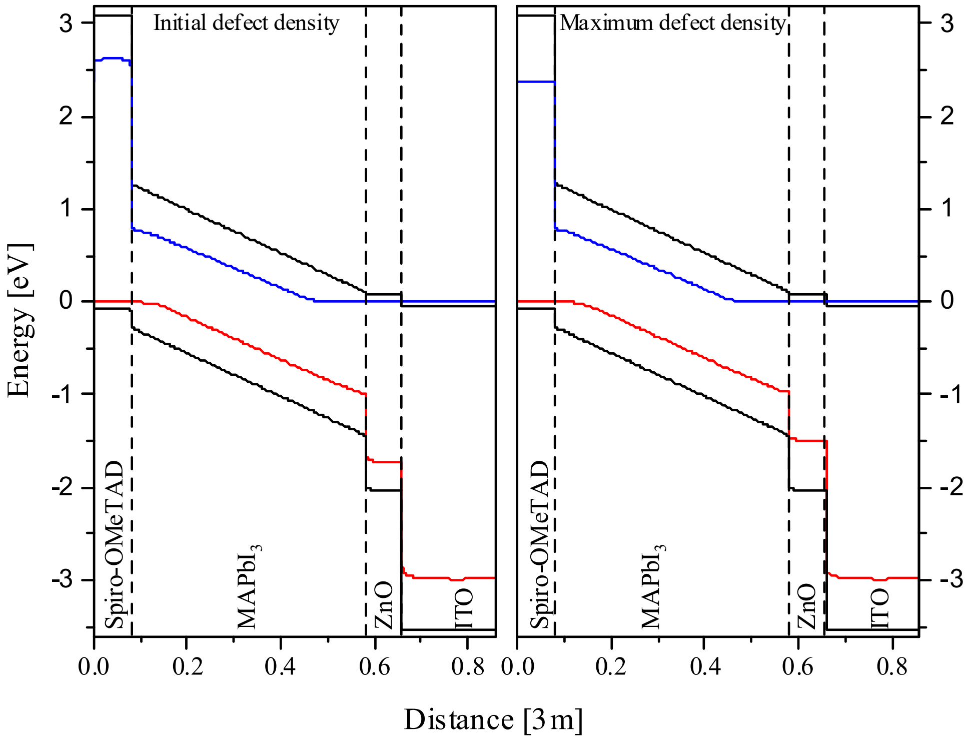

Figure S20. Influence of defect density on band structure of PSC.

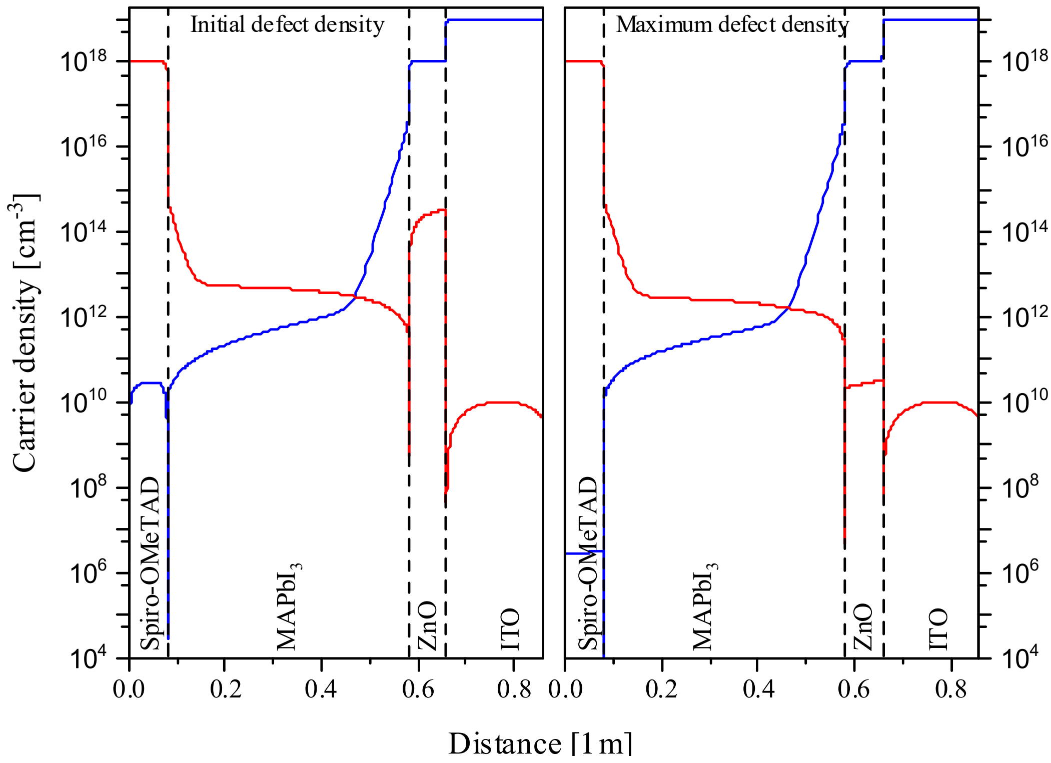

Figure S21. Influence of defect density on carrier density of PSC.

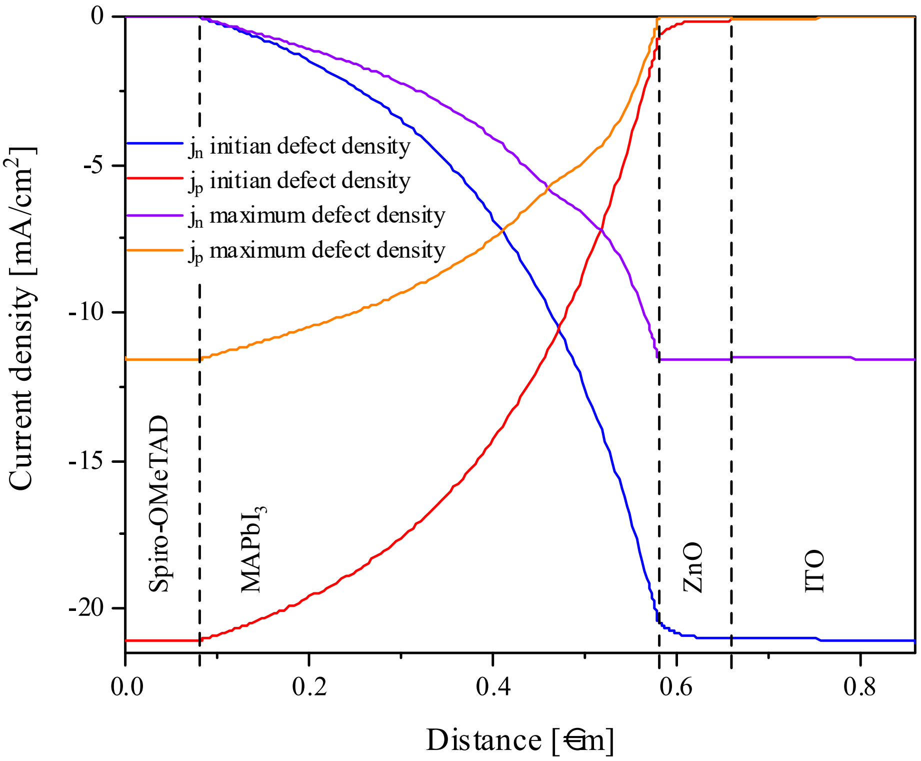

Figure S22. Influence of defect density on current density of PSC.

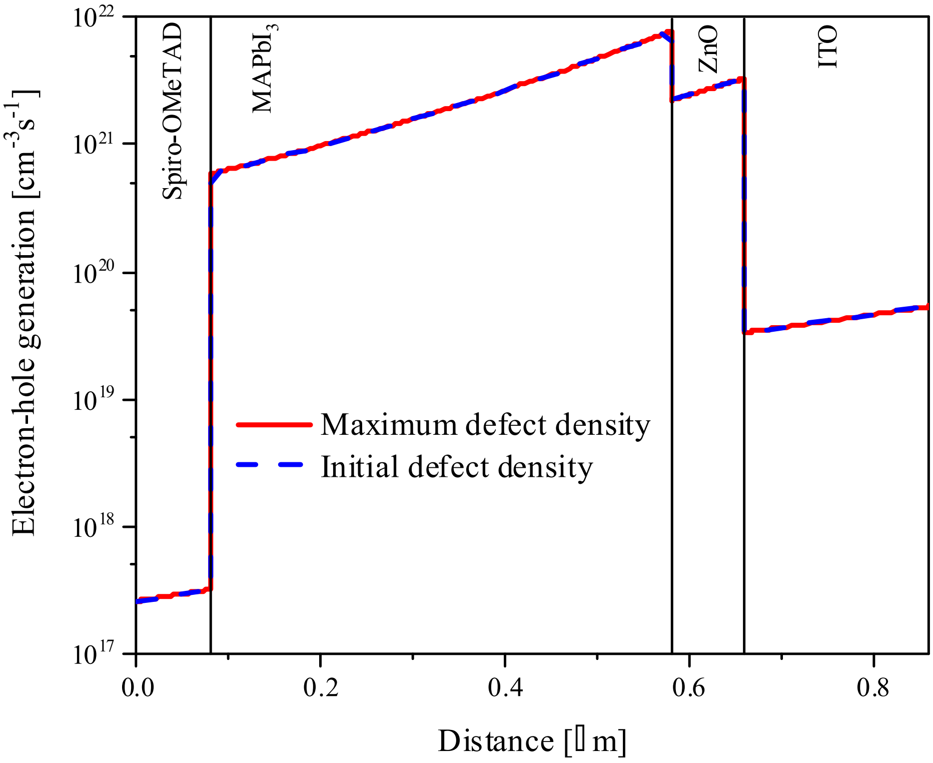

Figure S23. Influence of defect density on electron-hole generation function of PSC.

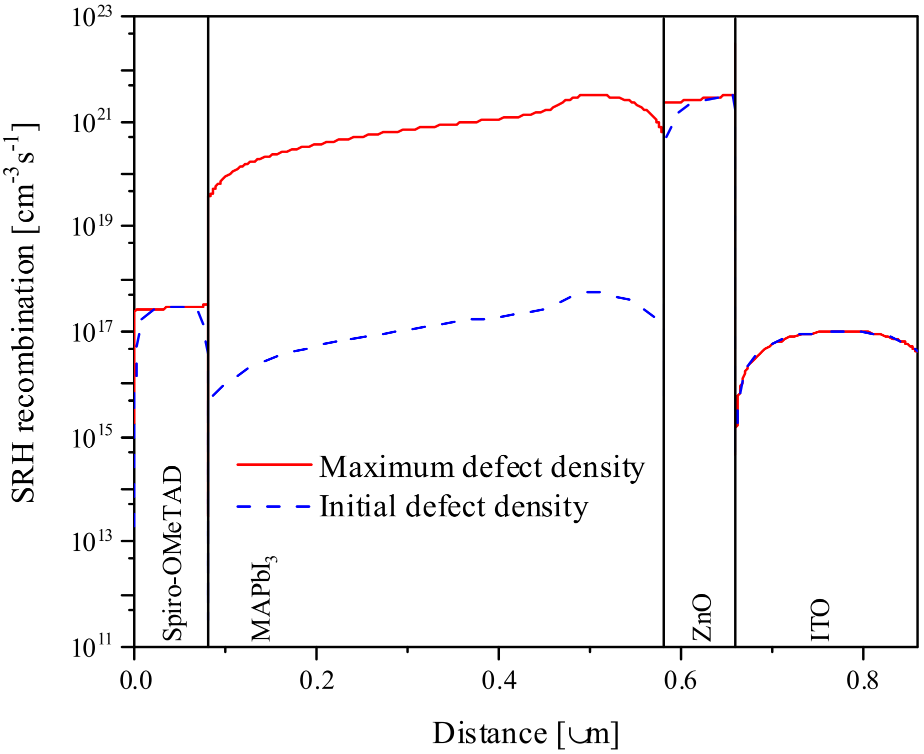

Figure S24. Influence of defect density on Shockley-Read-Hall recombination function of PSC.

Initial data for SCAPS modeling

Table S1. Initial material parameters used for charge transport layers [14-19].

Perovskite	Band gap, (eV)	Electron affinity (eV)	Dielectric permittivity	CB effective density of states (1/cm^3)	VB effective density of states (1/cm^3)	Electron mobility (cm^2/V×s)	Hole mobility (cm^2/V×s)	Shallow donor density (1/cm^3)	Shallow Acceptor density (1/cm^3)	Defect Density (1/cm^3	Electron thermal velocity (cm/s)	Hole thermal velocity (cm/s)
FTO	3.5	4	9	$2.2\cdot10^{18}$	$1.8\cdot10^{19}$	20	10	$1.0\cdot10^{19}$	0.00	$1.0\cdot10^{15}$	$1.0\cdot10^{7}$	$1.0\cdot10^{7}$
ZnO	3.3	4.1	9			100	25			$1.0\cdot10^{15}$	$1.0\cdot10^{7}$	$1.0\cdot10^{7}$
Spiro-omeTAD	3.17	2.1	3	$2.5\cdot10^{18}$	$1.8\cdot10^{19}$	$2.0\cdot10^{-4}$	$2.0\cdot10^{-4}$	0	$1.0\cdot10^{18}$	$1.0\cdot10^{15}$	$1.0\cdot10^{7}$	$1.0\cdot10^{7}$

Table S2. Initial perovskite material parameters [18-20].

Perovskite	Band gap, (eV)	Electron affinity (eV)	Dielectric permittivity	CB effective density of states (1/cm^3)	VB effective density of states (1/cm^3)	Electron mobility (cm^2/V×s)	Hole mobility (cm^2/V×s)	Shallow donor density (1/cm^3)	Shallow Acceptor density (1/cm^3)	Defect Density (1/cm^3	Electron thermal velocity (cm/s)	Hole thermal velocity (cm/s)
$MAPbBr_3$	2.3	3.9	6.5	$2.2\cdot10^{18}$	$1.8\cdot10^{19}$	20	20	0	0	$2.0\cdot10^{15}$	$1.0\cdot10^{7}$	$1.0\cdot10^{7}$
$CsPbI_3$	1.694	3.95	6	$1.1\cdot10^{20}$	$8.2\cdot10^{20}$	25	25	0	$1.0\cdot10^{15}$	$1.0\cdot10^{15}$	$1.0\cdot10^{7}$	$1.0\cdot10^{7}$
$FAPbI_3$	2.288	4	6.6	$1.2\cdot10^{19}$	$2.9\cdot10^{18}$	27	27	$1.3\cdot10^{16}$	$1.4\cdot10^{16}$	$4.0\cdot10^{13}$	$1.0\cdot10^{7}$	$1.0\cdot10^{7}$
$MAPbI_3$	1.547	3.9	10	$2.2\cdot10^{18}$	$1.8\cdot10^{19}$	2	1	$5.2\cdot10^{9}$	$5.2\cdot10^{9}$	$2.5\cdot10^{13}$	$1.0\cdot10^{7}$	$1.0\cdot10^{7}$
